# Quality of life, disability and antipsychotics-related side effects in schizophrenia spectrum and bipolar disorder: the role of patient-reported outcome measures

**DOI:** 10.1007/s00406-025-02169-8

**Published:** 2025-12-15

**Authors:** Sofia Francesca Aprile, Alessandro Rodolico, Sabrina Castellano, Pierfelice Cutrufelli, Antonio Di Francesco, Stefan Leucht, Filippo Caraci, Andrea Fiorillo, Maria Salvina Signorelli

**Affiliations:** 1 Department of Educational Sciences, Section of Psychology, University of Catania, Via Teatro Greco 84, 95124 Catania, Italy; 2 Department of Psychiatry and Psychotherapy, TUM School of Medicine and Health, Technical University of Munich, Klinikum rechts der Isar, Munich, Germany; 3 Psychiatry Unit, Department of Clinical and Experimental Medicine, University of Catania, Via Santa Sofia 78, 95123 Catania, Italy; 4 German Center for Mental Health, Berlin, Germany; 5 Department of Drug and Health Sciences, University of Catania, Viale Andrea Doria 6, 95123 Catania, Italy; 6 Oasi Research Institute, IRCCS, Via Conte Ruggero 73, 94018 Troina, Italy; 7 Department of Psychiatry, University of Campania “L. Vanvitelli”, Naples, Italy

**Keywords:** Antipsychotic, Side effects, Schizophrenia, Bipolar disorder, Quality of life, Network analysis

## Abstract

**Background:**

Schizophrenia and bipolar disorder significantly impair daily functioning and quality of life. Effective treatment monitoring requires tools assessing both clinical symptoms and everyday impairment.

**Aim:**

This cross-sectional study explored relationships between symptoms, side effects, and functioning using clinician-rated and patient-reported measures. We examined whether the Glasgow Antipsychotic Side-Effect Scale (GASS), a patient-reported outcome measure (PROM), is associated with disability and quality of life comparably to the clinician-administered Udvalg for Kliniske Undersøgelser (UKU) Side Effect Rating Scale.

**Methods:**

We recruited 100 individuals with schizophrenia spectrum or bipolar disorders receiving antipsychotic treatment. Linear regressions and network analyses were conducted. Linear regressions assessed the association between side effects, disability (World Health Organization Disability Assessment Schedule, WHODAS) and quality of life (EuroQol 5-Dimension scale, EQ-5D). Network analyses were conducted to explore the interplay between side effects, disability, quality of life and symptoms, the latter assessed using the Positive and Negative Syndrome Scale (PANSS), Young Mania Rating Scale (YMRS), and Montgomery–Åsberg Depression Rating Scale (MADRS).

**Results:**

Side effects assessed with GASS and UKU significantly impacted disability (GASS: *p* < .001, 95% CI [0.274; 0.635]; UKU: *p* < .001, 95% CI [0.313; 1.021]) and reduced quality of life (GASS: *p* < .001, 95% CI [-1.391; -0.511]; UKU: *p* < .001, 95% CI [-3.176; -1.668]). Network analysis identified depressive symptoms as central in the UKU network and autonomic side effects as central in the GASS network.

**Conclusion:**

GASS total and subgroup scores showed comparable associations with disability and quality of life as UKU. Integrating both perspectives enables comprehensive monitoring and patient-centred care.

**Supplementary Information:**

The online version contains supplementary material available at https://doi.org/10.1007/s00406-025-02169-8.

## Introduction

Schizophrenia and bipolar disorder are chronic psychiatric disorders that significantly impact individuals’ quality of life and daily functioning, being associated with considerable disability in personal, family, social, educational and occupational functioning. Antipsychotic medications are the recommended first-line treatment for both schizophrenia spectrum disorders and bipolar disorder in most International clinical guidelines [1]. However, these drugs are associated with a wide range of side effects that may further negatively impact patients’ quality of life and daily functioning [2]. A wide range of patient-reported outcome measures (PROMs) have been recently developed to evaluate these variables, along with other relevant factors related to symptoms and antipsychotics’ side effects. PROMs are valuable tools for evaluating treatment benefits and symptom improvement, emphasizing patients’ experiences of how they feel and function in relation to their health condition [3]. While patient-reported experience measures (PREMs) are focused on addressing the quality of experiences in care settings, PROMs capture patients’ perspectives on their symptoms, functional status and health-related quality of life [4]. The increasing emphasis on incorporating patients’ perspectives and experiences in clinical practice has led healthcare systems to recognize the value of PROMs, particularly in assessing patient satisfaction [5]. PROMs play a crucial role in integrating patients’ voices into routine clinical assessments, facilitating the evaluation of care quality, service management and policymaking in mental health. PROMs have been widely used in various clinical settings, and their importance has been increasingly recognized in mental health care [6, 7]. PROMs might predict outcomes in schizophrenia, such as hospitalization rates, and serve as essential tools for measuring quality of life and daily functioning [8]. Another important area where PROMs could enhance the management of psychotic disorders is the assessment of severity of adverse events. Traditionally, this evaluation has been conducted by psychiatrists using gold-standard tools, such as the UKU Side Effects Rating Scale [9]. The limited integration of patient-reported assessments in this domain has been attributed to the assumption that individuals with psychotic disorders lack insight into their condition, making their perspectives on treatment efficacy unreliable [10]. However, contemporary views on psychiatric illnesses are shifting, with organizations such as the International Consortium for Health Outcomes Measures (ICHOM) advocating for the development of self-reported tools for psychiatric patients [11].

The aim of this research is to investigate how standard PROMs might serve as valuable proxies for patient well-being. We employed the Glasgow Antipsychotics Side-Effects Scale (GASS), a self-report instrument designed to identify antipsychotic side effects [12], and to examine their interrelationship with patients’ functioning and quality of life. The main hypothesis is that a PROM measure is significantly associated with important domains for psychotic patients, such as quality of life and disability related to antipsychotic use, similar to clinician-led assessments. The secondary hypothesis is that, based on the network theory of psychopathology [13], PROMs may provide different insights compared to traditional clinician-administered tools for evaluating antipsychotic side effects.

## Method

### Participants

Inpatients and outpatients were recruited between September 2021 and April 2022 at the Psychiatry Unit of the “Policlinico Gaspare Rodolico” University Hospital in Catania, Italy. Participants included individuals diagnosed with schizophrenia spectrum disorders or bipolar disorders, according to the Diagnostic and Statistical Manual of Mental Disorders, Fifth Edition (DSM-5), who had been receiving antipsychotic treatment for at least four weeks. Inclusion and exclusion criteria have been described elsewhere [14] as this manuscript describes a secondary analysis paper, however they are briefly summarised as follows:

- Inclusion: adults (≥ 18) who are in- or outpatients with DSM-5 schizophrenia or bipolar spectrum disorder; on ≥ 1 antipsychotic for ≥ 6 months; currently without positive symptoms (PANSS p1/p3/g9 ≤ 3), without depression (MADRS < 10) or mania (YMRS < 7); good insight (PANSS g12 ≤ 3); no delusions/hallucinations if bipolar; able to understand questionnaires and the informed consent.
- Exclusion: compulsory treatment; organic diseases; current psychoactive substance use; neurological conditions (e.g., epilepsy, movement disorders, intellectual disability, dementia); or any condition hindering completion of the assessment.

The original purpose of the study was to validate the Italian version of the GASS scale, and the sample size was determined based on the recommended minimum requirements [15]. Psychometric tools were administered during routine check-up visits for outpatients and during hospitalization for inpatients. The authors assert that all procedures contributing to this work comply with the ethical standards of the relevant national and institutional committees on human experimentation and with the Helsinki Declaration of 1975, as revised in 2013. All procedures involving human patients were approved by the Institutional Review Board of the University of Catania (Protocol number: 1521). All subjects provided written informed consent for their scores to be used for research purposes. Data were collected in private rooms and securely stored in protected databases to maintain confidentiality. This observational, cross-sectional study was reported in accordance with the STROBE checklist (supplementary materials, Appendix E).

### Psychometric measures

#### Symptoms

Symptom measures were included to investigate their influence within the network structure. Psychotic symptoms were assessed using a subset of the Positive and Negative Syndrome Scale (PANSS) [16], a gold-standard clinician-rated tool for evaluating psychotic symptoms. For eligibility, we administered items related to symptom stability and insight, as defined by Andreasen [17] for remission (P1, P3, G9) and insight (G12).

Manic symptoms were assessed using the Young Mania Rating Scale (YMRS), an 11-item clinician-administered tool assessing mania severity [18]. Scores range from 0 to 60, with higher scores indicating greater severity.

Depressive symptoms were assessed using the Montgomery-Asberg Depression Rating Scale (MADRS) [19], a 10-item clinician-administered tool assessing depression severity. Scores range from 0 to 60, with higher scores indicating greater severity.

#### Disability and functioning

Functional disability was assessed using the Italian-validated, clinician-assisted 12-item World Health Organization Disability Assessment Schedule 2.0 (WHODAS) [20]. This tool evaluates functioning over the past 30 days across six domains (cognition, mobility, self-care, getting along, life activities, participation) using a 5-point visual analog scale (0 = no difficulty to 4 = extreme difficulty), aligned with the ICF framework.

#### Quality of life

Quality of life was assessed using the EuroQoL 5-Dimensions, 5-Levels (EQ-5D-5 L) scale [21]. For this study, only the visual analog scale (VAS) score, ranging from 0 (worst imaginable health) to 100 (best imaginable health), was used to represent overall perceived health status.

#### Antipsychotics side effects

Antipsychotic side effects were assessed using the Italian-validated Glasgow Antipsychotic Side-Effects Scale (GASS) and the UKU Side Effects Rating Scale.

The GASS, a 22-item self-report scale assessing side effects over the past two weeks (Waddell & Taylor, 2008), uses a 0–3 frequency Likert scale and a 1–10 distress scale for each item. Its Italian validation showed a one-factor structure [14], but for clinical utility, items were grouped into psychological, neurological, autonomic, and sexual subcategories based on the UKU classification [2].

The UKU [9], a 48-item clinician-administered scale (0–3 severity Likert), is a gold standard for psychotropic drug side effects [22]. Items are categorized into psychological, neurological, autonomic, and other adverse effects. Sexual adverse effects (UKU items 4.7–4.16) were analysed separately for detailed insights. Total and subscale scores are sums of item severity ratings.

### Data analysis

Quantitative variables were analysed on their continuous scales, and because no data were missing, imputation was not required. Statistical analyses and psychometric network analyses were performed using the open-source software JASP (2024).

#### Inferential analyses

Associations between disability, quality of life, and each scale were examined using linear regression. A simple linear regression model was first applied, where a single explanatory variable (x) and a response variable (y) are described using the equation:

$${\mathrm{y}} = \beta _{0} + \beta _{{1X}} + \in ,$$

with β_0_ (intercept) and β_1_ (slope) as unknown constants, and ε as the random error term [23, 24]. In this model, side effects measured via the GASS and UKU scales served as explanation of disability and quality of life outcomes.

All statistical tests used a significance level of α = 0.05. Bonferroni correction was not applied, as each hypothesis was tested independently, avoiding an unnecessary increase in Type II errors [25]. Finally, internal reliability of the WHODAS, GASS, UKU, YMRS, MADRS scores was verified using Cronbach’s alpha and McDonald’s omega (supplementary materials, Appendix A). Internal reliability could not be assessed for the EQ-5D-5 L because only the VAS index was collected in this study.

#### Network analysis

The use of network analysis arises from the recognition that mental disorders are complex, multifaceted conditions characterized by intricate, interrelated factors. This approach acknowledges the dynamic interactions and reciprocal influences among various variables in psychiatric research, providing a more holistic understanding of psychopathology. Network models offer a valuable approach to bridging the existing gap in clinical research, which often relies on a reductionist perspective of mental illness [26]. According to network approaches, there is no single cause for psychopathology, but mental disorders are caused by the dynamic interaction between symptoms [27]. These networks are then visually represented as nodes and edges, where nodes correspond to variables, and edges (links between nodes) represent interactions between them. Each edge specifically denotes a pairwise interaction (k = 2). Since all variables in this study were continuous, Gaussian Graphical Models (GGM) were used to visually represent networks [28]. For regularization, the graphical least absolute shrinkage and selection operator (Glasso) with the extended Bayesian information criterion (EBIC) for network estimation was used [29]. The Glasso method applies a tuning parameter (λ = 0.5) regularization to estimate sparse inverse covariance matrices, making it particularly effective for high-dimensional data [30]. EBIC was used for model selection, as it helps determine the optimal level of regularization by balancing model complexity and fit [31]. The total sample was used to generate two different networks, both of which included the following variables: disability, quality of life, symptoms, and side effects. In the first network, side effects were assessed using GASS, while in the second they were assessed using the UKU scale. The two networks were then visually compared to explore differences when the same variable was measured through a PROM (GASS) versus a clinician-administered gold-standard tool (UKU). Networks were compared using centrality measures, including strength, closeness and betweenness. Strength refers to the sum of the weights of all edges connected to a node. Closeness is the inverse of the sum of a node’s distances from all other nodes in the network, indicating how easily a node can influence others. Betweenness represents the number of shortest paths between any two nodes that pass through the focal node, reflecting its role as a bridge within the network. Centrality measures are useful for assessing the extent to which a specific variable is interconnected with others in the network [32]. In addition, expected influence was calculated. While centrality measures focus on a node’s position in the network, expected influence identifies the overall impact and influence of a node, and it is considered important in identifying key nodes in mental disorders networks [33].

The author(s) made use of ChatGPT-4o, accessed from OpenAI (https://openai.com/chatgpt), to assist with the drafting of this article. The authors reviewed and edited the content as needed and took full responsibility for the content of the article.

## Results

### Sociodemographic and clinical characteristics of the sample

The total sample included 100 participants, 61 being males and 39 females. The mean age was 45.4 (SD = 12.4), ranging from 19 to 71 years. 69 patients had a diagnosis of schizophrenia spectrum disorders and 31 of bipolar disorder. Relevant characteristics of the sample are reported in supplementary materials (Table S1).

### Linear regression analyses

Regression analyses were conducted to examine whether side effects, and which specific ones, are associated with disability and quality of life. Each coefficient represents the expected change on the WHODAS or EQ-5D-5 L scale per one-point increase on the respective GASS/UKU side-effect score, whose items use 0–3 ratings. Significant results are reported in Table 1; complete data analysis with confidence intervals are reported in supplementary materials (Appendix B).

**Table 1:** Linear regression analyses for WHODAS and EQ-5D-5 L

WHODAS						
			ANOVA			
Variable	R^**2**^	Adjusted R^**2**^	F	p	B (SE)	t
GASS overall	0.203	0.195	24.906	< 0.001	0.454 (0.091)	4.991
GASS autonomic SE	0.088	0.079	9.514	0.003	0.612 (0.297)	3.084
GASS neurological SE	0.223	0.215	28.101	< 0.001	1.330 (0.471)	5.301
GASS psychological SE	0.135	0.126	15.254	< 0.001	2.200 (0.563)	3.906
UKU overall	0.125	0.116	13.986	< 0.001	0.667 (0.353)	3.740
UKU psychological SE	0.157	0.148	18.223	< 0.001	3.752 (0.879)	4.269
UKU neurological SE	0.130	0.121	14.651	< 0.001	3.653 (0.954)	3.828

EQ-5D-5 L						
			ANOVA			
Variable	R^**2**^	Adjusted R^**2**^	F	p	B (SE)	t
GASS overall	0.158	0.150	18.418	< 0.001	− 0.951 (0.222)	− 4.292
GASS psychological SE	0.112	0.103	12.392	< 0.001	− 4.762 (1.353)	− 3.520
GASS neurological SE	0.118	0.109	13.092	< 0.001	− 2.292 (0.634)	− 3.618
GASS autonomic SE	0.136	0.127	15.458	< 0.001	− 1.799 (0.458)	− 3.932
UKU overall	0.293	0.286	40.614	< 0.001	− 2.422 (0.380)	− 6.373
UKU neurological SE	0.213	0.205	26.509	< 0.001	− 11.082 (2.152)	− 5.149
UKU psychological SE	0.207	0.199	25.531	< 0.001	− 10.214 (2.021)	− 5.053
UKU autonomic SE	0.143	0.134	16.359	< 0.001	− 3.763 (0.930)	− 4.045
UKU sexual SE	0.083	0.074	8.883	0.004	− 2.626 (0.881)	− 2.980

Side effects identified using both the UKU (*p* <.001) and GASS (*p* <.001) scales were significantly associated with disability, with higher side-effect scores being associated with greater disability. Among specific side effects, autonomic (*p* =.003), psychological (*p* <.001), and neurological side effects (*p* <.001) were significant when measured with the GASS scale, whereas psychological (*p* <.001) and neurological (*p* <.001) side effects emerged as significant with the UKU scale. Side effects identified using both the UKU (*p* <.001) and GASS (*p* <.001) scales were significantly associated with quality of life. Among specific side effects, autonomic, psychological and neurological side effects were significant with the GASS scale, whereas psychological and neurological side effects emerged as significant using the UKU scale.

### Network analysis

**Fig. 1:**
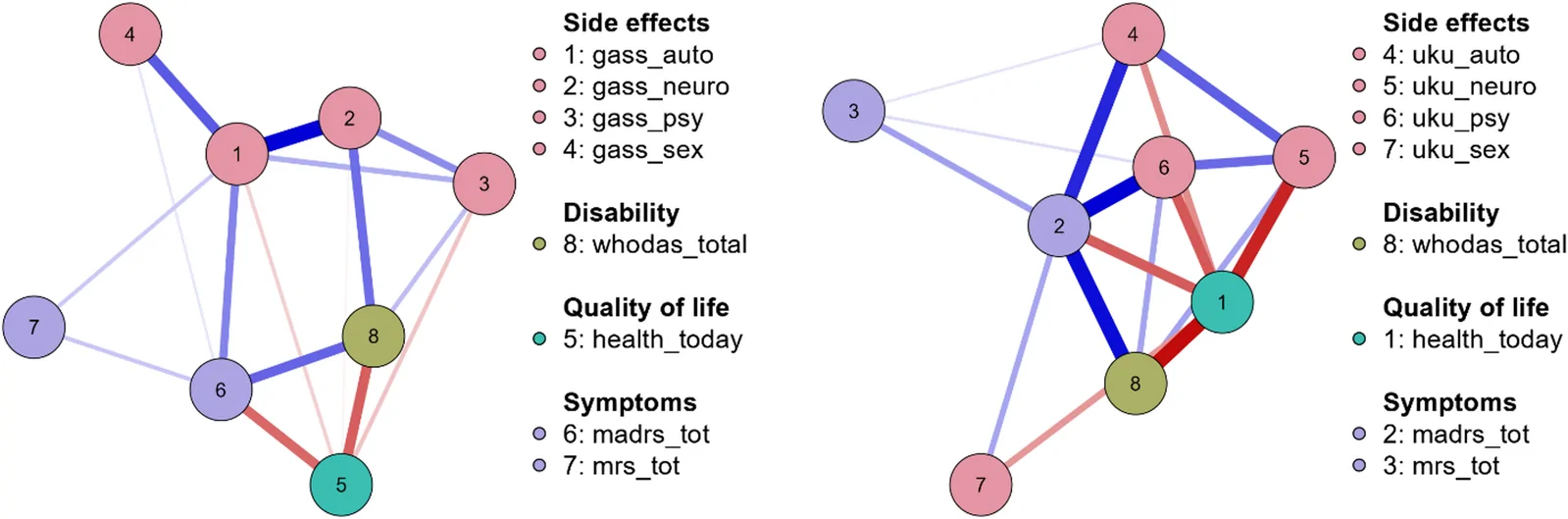
Interactions between disability, quality of life, symptoms and side effects using GASS (N1) versus disability, quality of life, symptoms and side effects using UKU (N2)^a^. ^a^gass_psy: GASS psychological SE; gass_neuro: GASS neurological SE; gass_auto: GASS autonomic SE; gass_sex: GASS sexual SE; whodas_total: WHODAS overall score; health_today: EQ-5D VAS score; madrs_tot: MADRS total score; mrs_tot: YMRS total score; whodas_total: WHODAS total score; health_today: EQ-5D VAS score; mrs_tot: YMRS overall score; madrs_tot: MADRS overall score; uku_psy: UKU psychological SE; uku_neuro: UKU neurological SE; uku_auto: UKU autonomic SE; uku_sex: UKU sexual S

Figure 1 displays two distinct network plots illustrating the interactions between disability, quality of life, and side effects. Network 1 (N1) explored side effects assessed by the GASS, while Network 2 (N2) used the UKU for side effect assessment. Centrality measures for both networks are detailed in Table 2.

N1 resulted in a dense network (non-zero edges 57.1%; sparsity 0.43). GASS autonomic side effects was the most robust node (strength = 1.526; expected influence = 1.371). YMRS (mrs_tot) showed the highest absolute value of strength (−1.530), but its negative values suggest it might have an inverse impact in the network. Quality of life (health_today) had the highest negative value of expected influence (− 2.024). N2 showed a dense network (non-zero edges = 60.7%; sparsity = 0.393). MADRS represented the most influential node with high strength (1.351) and expected influence (1.246). EQ-5D-5 L showed the highest absolute value with a negative expected influence (− 2.227).

**Table 2:** Centrality measures and expected influence for N1 and N2

N1				
Variable	Betweenness	Closeness	Strength	Expected influence
GASS psychological SE	− 0.610	− 0.885	− 0.489	− 0.003
GASS neurological SE	− 0.036	1.052	0.549	0.967
GASS autonomic SE	2.262	1.216	1.526	1.371
GASS sexual SE	− 0.610	− 0.516	− 1.129	− 0.039
WHODAS	− 0.323	0.491	0.548	0.008
EQ-5D-5 L	− 0.610	− 0.226	0.064	− 2.024
MADRS	0.538	0.537	0.461	0.017
YMRS	− 0.610	− 1.669	− 1.530	− 0.297

N2				
Variable	Betweenness	Closeness	Strength	Expected influence
UKU psychological SE	0.000	0.361	0.244	0.499
UKU neurological SE	− 0.524	− 0.196	0.015	0.139
UKU autonomic SE	− 0.524	0.051	− 0.291	0.358
UKU sexual SE	− 0.524	− 1.280	− 1.283	− 0.250
WHODAS	− 0.524	0.417	0.163	0.145
EQ-5D-5 L	0.262	0.889	1.193	− 2.227
MADRS	2.358	1.327	1.351	1.246
YMRS	− 0.524	− 1.570	− 1.391	0.090

An exploratory analysis of symptoms was conducted using individual GASS items and is presented in supplementary materials (Appendix C).

## Discussion

This study explored the impact of PROMs in clinical practice for assessing antipsychotic side effects and evaluating drug efficacy. The objective was to highlight the usefulness of self-reported instruments in routine clinical settings, particularly in monitoring treatment outcomes and treatment-related aspects. The GASS and UKU scales were used together in a cross-sectional sample of patients diagnosed with schizophrenia spectrum and bipolar disorders to assess side effects. GASS items were divided into subgroups following the UKU classification. Data analyses showed that not only the overall GASS score but also its subscores were significantly associated with disability and quality of life, comparable to the UKU scale. However, while only the psychological and neurological UKU subscores were significantly associated with the WHODAS score, autonomic, neurological, and psychological side effects assessed through GASS were significantly associated with disability. Both scales were also significantly associated with quality of life. These results suggest that patient-reported outcome measures (PROMs), such as the GASS scale, may be as useful in clinical practice as traditional clinician-administered tools. This aligns with existing literature advocating for a more central role of the patient in psychiatric care [34, 35]. Qualitative research has also been conducted on themes related to drug treatment options and side effects, aiming to better understand patients’ perspectives and inform more patient-centred approaches to care [36, 37]. Moreover, although no standardized subscales exist for the GASS scale, clinicians might consider using its sub scores to better understand the impact of antipsychotics on daily life. Notably, quality of life is often treated as a secondary outcome in randomized clinical trials (RCTs), yet it plays a crucial role in defining patient-tailored treatment approaches [38].

Network analysis was then used to visually represent the dynamic interactions between the measured variables, that are represented as nodes (one for each variable) and edges (connections between them). N1 and N2 represented the same constructs; however, while N1 reflected patient-reported side effects, N2 captured clinician-assessed adverse events, as typically conducted in psychiatric clinical practice. Centrality measures revealed differences between them. The centrality analysis of N1 highlights key variables influencing the network of patient-reported side effects (GASS-based measures). The strongest and most influential node is GASS autonomic side effects, indicating that autonomic symptoms are highly interconnected with other factors in the network. This suggests that autonomic side effects may play a crucial role in shaping the overall experience of antipsychotic-related side effects. The robustness of this node might indicate that autonomic symptoms are not isolated but interact with multiple dimensions of functioning, potentially worsening disability or perceived well-being. Other relevant nodes are mania symptoms and quality of life. The negative values of YMRS suggest that higher mania symptoms are linked to a lower impact of other variables, potentially indicating an attenuated perception of side effects in manic states. Quality of life is strongly affected by side effects, with negative values indicating that worse quality of life is associated with a greater impact of other side effects. This highlights the importance of monitoring patient-reported outcomes. The second network (N2) showed crucial differences when compared to N1. Depressive symptoms (MADRS) represented the most robust node, playing a major role in the network and thus suggesting a possible exacerbation of disability and quality of life. Quality of life showed strong negative expected influence, thus suggesting a high sensitivity to other symptoms and side effects. As in N1, manic symptoms (YMRS) seem to have a dampening effect on the perception of side effects. Finally, neurological and autonomic side effects have minimal influence in this network. The most notable difference lies in the completely different positioning of side effects within the two networks: they are central when assessed by patients but become peripheral when evaluated by clinicians. These results are in line with available literature enhancing the differences between clinician-assessed and patient-reported adverse events, thus suggesting that incorporating patient perspective might enhance the monitoring of subjective side effects, leading to more timely and accurate evaluations and ultimately improving patient-centred care [39].

### Limitations and strengths

A first limitation of this study is the relatively small sample size. Although larger samples are generally recommended for more robust conclusions, prior research indicates that even modest sample sizes (e.g., around 100 observations) can reliably identify the strongest network connections [40]. There is no universally accepted minimum sample size for estimating Gaussian Graphical Models (GGMs) with continuous data, as adequacy depends on factors such as the number of nodes, expected sparsity, and strength of associations [30]. Heuristic guidelines from regression modeling, such as the “one in ten” rule, suggest that a sample size of approximately 100 may be sufficient for networks with about ten nodes. In our case, networks appeared particularly dense, which, along with the use of the standard EBIC GLasso tuning parameter (λ = 0.5), supports the reliability of our results despite the sample size. Nonetheless, larger samples would enhance the generalizability and stability of the findings. While this limitation should be acknowledged, several methodological and contextual strengths counterbalance them. Another limitation of this study is that it was conducted at a single center. Multicenter studies are needed to address this limitation and provide more generalizable insights.

One notable strength is the use of a real-world outpatient clinical sample, which enhances the ecological validity and applicability of the findings to routine practice. Although this introduces heterogeneity, such diversity reflects the complexity of everyday clinical settings and increases the relevance of the results across a broad patient population. Additionally, the inclusion of patients with schizophrenia and bipolar disorder is supported by evidence that quality of life is similarly impaired in both groups [41], reducing the likelihood that diagnosis acted as a confounding variable. Another strength lies in the focus on antipsychotic-induced side effects, which have been consistently associated with lower quality of life in larger cross-sectional studies [42]. Lastly, the dense structure of the estimated networks, retained under standard tuning parameters, likely enhances signal detection in smaller samples, further supporting the robustness of our network estimations. A key limitation is that, while we used PROMs for quality of life, disability, and side effects, we did not include patient-reported symptom measures (e.g., those recommended by the ICHOM consortium). This reflects the limited availability of brief, psychometrically robust self-report scales for core psychotic symptoms and concerns about construct comparability with clinician-rated instruments; consequently, symptom nodes are represented only by clinician-rated measures, which may underrepresent patients’ subjective symptom burden and constrain cross-method convergence in the network. Future studies should incorporate validated symptom PROMs to triangulate domains and examine whether network structure is consistent across reporter types.

### Conclusion

This study highlights the potential of PROMs, specifically the GASS scale, as a valuable tool for assessing antipsychotic side effects in psychiatric clinical practice. Findings support the primary hypothesis that PROMs might effectively be associated with key domains such as quality of life and disability, comparable to the clinician-administered UKU scale. The significant associations emerging from the GASS total and subscale scores reinforces its clinical utility in capturing the impact of side effects on patients’ disability in dailylife. While clinician-administered tools remain the gold standard, patient-reported measures provide unique and complementary insights, as suggested by network theory. Network analysis revealed distinct differences between patient- and clinician-reported outcomes. In the patient-reported network (N1), autonomic side effects were the most influential, indicating their strong interconnection with other symptoms and their role in shaping treatment experiences. Manic symptoms appeared to dampen the perception of side effects, while quality of life was significantly impacted. Conversely, the clinician-assessed network (N2) was dominated by depressive symptoms, with autonomic and neurological side effects playing a minimal role. This contrast underscores the differences in how side effects are perceived and prioritized, suggesting that integrating PROMs into clinical practice could improve the monitoring of subjective side effects often overlooked by clinicians. Overall, these findings support the integration of PROMs as GASS into psychiatric settings to complement clinician assessments, enhance patient-centred care, and improve treatment personalization. Future research should focus on refining PROM subscales and exploring their role in longitudinal monitoring of treatment outcomes.

## Supplementary Information

Below is the link to the electronic supplementary material.


Supplementary Material 1


## Data Availability

The data that support the findings of this study are available from the corresponding author, F.C., upon reasonable request.
