# Supplementary material for: Quality of life, disability and antipsychotics-related side effects in schizophrenia spectrum and bipolar disorder: the role of patient-reported outcome measures
Source: Eur Arch Psychiatry Clin Neurosci. 2025 Dec 15;276(6):2861–9. doi: 10.1007/s00406-025-02169-8 (PMC13526042; doi:10.1007/s00406-025-02169-8)
Supplement: Supplementary file 1 [file 406_2025_2169_MOESM1_ESM.docx]

**Supplementary materials**

**Appendix A**

**Table S1**. Sample characteristics

| **Characteristics** | **Category** | **Total sample N = 100** |
| --- | --- | --- |
| Age | Mean (SD) [range] | 45.4 (12.4) [19-71] |
| Gender | Males  Females | 61 (61.0%)  39 (39.0%) |
| Education | Illiterate  Primary school  Middle school  High school  University degree | 1 (1.0%)  9 (9.0%)  35 (35.0%)  43 (43.0%)  12 (12.0%) |
| Occupation | No occupation  Worker | 75 (75.0%)  25 (25.0%) |
| Marital status | Maiden/divorced  Married | 72 (72.0%)  28 (28.0%) |
| Parental status | With children  Without children | 37 (37.0%)  63 (63.0%) |
| Smoking | Yes  No | 50 (50.0%)  50 (50.0%) |
| Diagnosis | Schizophrenia spectrum  Bipolar disorder | 69 (69.0%)  31 (31.0%) |
| Family history of psychiatric disorders | Yes  No | 42 (42.0%)  58 (58.0%) |
| Dysthyroid | Yes  No | 9 (9.0%)  91 (91.0%) |
| Diabetes | Yes  No | 8 (8.0%)  92 (92.0%) |
| Hypertension | Yes  No | 14 (14.0%)  86 (86.0%) |
| Hypercholesterolemia | Yes  No | 7 (7.0%)  93 (93.0%) |
| Antipsychotic treatment | Monotherapy  Polypharmacy | 71 (71.0%)  29 (29.0%) |
| Antipsychotic administration | Oral  LAI | 71 (71.0%)  29 (29.0%) |
| Antipsychotic group* | First generation  Second generation  Third generation | 4 (4.0%)  72 (72.0%)  24 (24.0%) |
| Antipsychotic dosage (mg/die) | Olanzapine equivalent, mean (SD) | 12.7 (6.94) |

*First-generation: promazine, zuclopenthixol, clotiapine; Second-generation: clozapine, risperidone, olanzapine, paliperidone, quetiapine; third-generation: aripiprazole, lurasidone, brexpiprazole, cariprazine. For those receiving polypharmacy, the antipsychotic with the highest dosage was considered.

Table S2. Internal reliability measures for GASS, UKU, WHODAS, YMRS, MADRS scales

| **Scale/subscale** | **Internal reliability** | **N** |
| --- | --- | --- |
| GASS | α = 0.810  ω = 0.804 | 100 |
| UKU | α = 0.676  ω = 0.674 | 100 |
| WHODAS | α = 0.896  ω = 0.901 | 100 |
| YMRS | α = 0.619  ω = 0.656 | 100 |
| MADRS | α = 0.862  ω = 0.871 | 100 |

**Appendix B**

**Linear regression analyses for disability (WHODAS)**

**GASS total score**

| *Model Summary - whodas_total* | | | | | | | | | |
| --- | --- | --- | --- | --- | --- | --- | --- | --- | --- |
| Model | | R | | R² | | Adjusted R² | | RMSE | |
| M₀ |  | 0.000 |  | 0.000 |  | 0.000 |  | 9.715 |  |
| M₁ |  | 0.450 |  | 0.203 |  | 0.195 |  | 8.719 |  |
|  | | | | | | | | | |
| *Note.*  M₁ includes gass_total | | | | | | | | | |

| *ANOVA* | | | | | | | | | | | | | |
| --- | --- | --- | --- | --- | --- | --- | --- | --- | --- | --- | --- | --- | --- |
| Model | |  | | Sum of Squares | | df | | Mean Square | | F | | p | |
| M₁ |  | Regression |  | 1893.307 |  | 1 |  | 1893.307 |  | 24.906 |  | < .001 |  |
|  |  | Residual |  | 7449.693 |  | 98 |  | 76.017 |  |  |  |  |  |
|  |  | Total |  | 9343.000 |  | 99 |  |  |  |  |  |  |  |
|  | | | | | | | | | | | | | |
| *Note.*  M₁ includes gass_total | | | | | | | | | | | | | |
| *Note.*  The intercept model is omitted, as no meaningful information can be shown. | | | | | | | | | | | | | |

| *Coefficients* | | | | | | | | | | | | | | | | | |
| --- | --- | --- | --- | --- | --- | --- | --- | --- | --- | --- | --- | --- | --- | --- | --- | --- | --- |
|  | | | | | | | | | | | | | | 95% CI | | | |
| Model | |  | | Unstandardized | | Standard Error | | Standardized | | t | | p | | Lower | | Upper | |
| M₀ |  | (Intercept) |  | 23.100 |  | 0.971 |  |  |  | 23.779 |  | < .001 |  | 21.172 |  | 25.028 |  |
| M₁ |  | (Intercept) |  | 16.632 |  | 1.562 |  |  |  | 10.649 |  | < .001 |  | 13.533 |  | 19.732 |  |
|  |  | gass_total |  | 0.454 |  | 0.091 |  | 0.450 |  | 4.991 |  | < .001 |  | 0.274 |  | 0.635 |  |
|  | | | | | | | | | | | | | | | | | |

**GASS psychological side effects**

| *Model Summary - whodas_total* | | | | | | | | | |
| --- | --- | --- | --- | --- | --- | --- | --- | --- | --- |
| Model | | R | | R² | | Adjusted R² | | RMSE | |
| M₀ |  | 0.000 |  | 0.000 |  | 0.000 |  | 9.715 |  |
| M₁ |  | 0.367 |  | 0.135 |  | 0.126 |  | 9.083 |  |
|  | | | | | | | | | |
| *Note.*  M₁ includes gass_psy | | | | | | | | | |

| *ANOVA* | | | | | | | | | | | | | |
| --- | --- | --- | --- | --- | --- | --- | --- | --- | --- | --- | --- | --- | --- |
| Model | |  | | Sum of Squares | | df | | Mean Square | | F | | p | |
| M₁ |  | Regression |  | 1258.400 |  | 1 |  | 1258.400 |  | 15.254 |  | < .001 |  |
|  |  | Residual |  | 8084.600 |  | 98 |  | 82.496 |  |  |  |  |  |
|  |  | Total |  | 9343.000 |  | 99 |  |  |  |  |  |  |  |
|  | | | | | | | | | | | | | |
| *Note.*  M₁ includes gass_psy | | | | | | | | | | | | | |
| *Note.*  The intercept model is omitted, as no meaningful information can be shown. | | | | | | | | | | | | | |

| *Coefficients* | | | | | | | | | | | | | | | | | |
| --- | --- | --- | --- | --- | --- | --- | --- | --- | --- | --- | --- | --- | --- | --- | --- | --- | --- |
|  | | | | | | | | | | | | | | 95% CI | | | |
| Model | |  | | Unstandardized | | Standard Error | | Standardized | | t | | p | | Lower | | Upper | |
| M₀ |  | (Intercept) |  | 23.100 |  | 0.971 |  |  |  | 23.779 |  | < .001 |  | 21.172 |  | 25.028 |  |
| M₁ |  | (Intercept) |  | 18.700 |  | 1.447 |  |  |  | 12.922 |  | < .001 |  | 15.828 |  | 21.572 |  |
|  |  | gass_psy |  | 2.200 |  | 0.563 |  | 0.367 |  | 3.906 |  | < .001 |  | 1.082 |  | 3.318 |  |
|  | | | | | | | | | | | | | | | | | |

**GASS neurological side effects**

| *Model Summary - whodas_total* | | | | | | | | | |
| --- | --- | --- | --- | --- | --- | --- | --- | --- | --- |
| Model | | R | | R² | | Adjusted R² | | RMSE | |
| M₀ |  | 0.000 |  | 0.000 |  | 0.000 |  | 9.715 |  |
| M₁ |  | 0.472 |  | 0.223 |  | 0.215 |  | 8.608 |  |
|  | | | | | | | | | |
| *Note.*  M₁ includes gass_neuro | | | | | | | | | |

| *ANOVA* | | | | | | | | | | | | | |
| --- | --- | --- | --- | --- | --- | --- | --- | --- | --- | --- | --- | --- | --- |
| Model | |  | | Sum of Squares | | df | | Mean Square | | F | | p | |
| M₁ |  | Regression |  | 2082.042 |  | 1 |  | 2082.042 |  | 28.101 |  | < .001 |  |
|  |  | Residual |  | 7260.958 |  | 98 |  | 74.091 |  |  |  |  |  |
|  |  | Total |  | 9343.000 |  | 99 |  |  |  |  |  |  |  |
|  | | | | | | | | | | | | | |
| *Note.*  M₁ includes gass_neuro | | | | | | | | | | | | | |
| *Note.*  The intercept model is omitted, as no meaningful information can be shown. | | | | | | | | | | | | | |

| *Coefficients* | | | | | | | | | | | | | | | | | |
| --- | --- | --- | --- | --- | --- | --- | --- | --- | --- | --- | --- | --- | --- | --- | --- | --- | --- |
|  | | | | | | | | | | | | | | 95% CI | | | |
| Model | |  | | Unstandardized | | Standard Error | | Standardized | | t | | p | | Lower | | Upper | |
| M₀ |  | (Intercept) |  | 23.100 |  | 0.971 |  |  |  | 23.779 |  | < .001 |  | 21.172 |  | 25.028 |  |
| M₁ |  | (Intercept) |  | 18.286 |  | 1.251 |  |  |  | 14.615 |  | < .001 |  | 15.804 |  | 20.769 |  |
|  |  | gass_neuro |  | 1.330 |  | 0.251 |  | 0.472 |  | 5.301 |  | < .001 |  | 0.832 |  | 1.827 |  |
|  | | | | | | | | | | | | | | | | | |

**GASS autonomic side effects**

| *Model Summary - whodas_total* | | | | | | | | | |
| --- | --- | --- | --- | --- | --- | --- | --- | --- | --- |
| Model | | R | | R² | | Adjusted R² | | RMSE | |
| M₀ |  | 0.000 |  | 0.000 |  | 0.000 |  | 9.715 |  |
| M₁ |  | 0.297 |  | 0.088 |  | 0.079 |  | 9.322 |  |
|  | | | | | | | | | |
| *Note.*  M₁ includes gass_auto | | | | | | | | | |

| *ANOVA* | | | | | | | | | | | | | |
| --- | --- | --- | --- | --- | --- | --- | --- | --- | --- | --- | --- | --- | --- |
| Model | |  | | Sum of Squares | | df | | Mean Square | | F | | p | |
| M₁ |  | Regression |  | 826.751 |  | 1 |  | 826.751 |  | 9.514 |  | 0.003 |  |
|  |  | Residual |  | 8516.249 |  | 98 |  | 86.900 |  |  |  |  |  |
|  |  | Total |  | 9343.000 |  | 99 |  |  |  |  |  |  |  |
|  | | | | | | | | | | | | | |
| *Note.*  M₁ includes gass_auto | | | | | | | | | | | | | |
| *Note.*  The intercept model is omitted, as no meaningful information can be shown. | | | | | | | | | | | | | |

| *Coefficients* | | | | | | | | | | | | | | | | | |
| --- | --- | --- | --- | --- | --- | --- | --- | --- | --- | --- | --- | --- | --- | --- | --- | --- | --- |
|  | | | | | | | | | | | | | | 95% CI | | | |
| Model | |  | | Unstandardized | | Standard Error | | Standardized | | t | | p | | Lower | | Upper | |
| M₀ |  | (Intercept) |  | 23.100 |  | 0.971 |  |  |  | 23.779 |  | < .001 |  | 21.172 |  | 25.028 |  |
| M₁ |  | (Intercept) |  | 19.467 |  | 1.502 |  |  |  | 12.959 |  | < .001 |  | 16.486 |  | 22.448 |  |
|  |  | gass_auto |  | 0.612 |  | 0.198 |  | 0.297 |  | 3.084 |  | 0.003 |  | 0.218 |  | 1.005 |  |
|  | | | | | | | | | | | | | | | | | |

**GASS sexual side effects**

| *Model Summary - whodas_total* | | | | | | | | | |
| --- | --- | --- | --- | --- | --- | --- | --- | --- | --- |
| Model | | R | | R² | | Adjusted R² | | RMSE | |
| M₀ |  | 0.000 |  | 0.000 |  | 0.000 |  | 9.715 |  |
| M₁ |  | 0.184 |  | 0.034 |  | 0.024 |  | 9.598 |  |
|  | | | | | | | | | |
| *Note.*  M₁ includes gass_sex | | | | | | | | | |

| *ANOVA* | | | | | | | | | | | | | |
| --- | --- | --- | --- | --- | --- | --- | --- | --- | --- | --- | --- | --- | --- |
| Model | |  | | Sum of Squares | | df | | Mean Square | | F | | p | |
| M₁ |  | Regression |  | 315.441 |  | 1 |  | 315.441 |  | 3.424 |  | 0.067 |  |
|  |  | Residual |  | 9027.559 |  | 98 |  | 92.118 |  |  |  |  |  |
|  |  | Total |  | 9343.000 |  | 99 |  |  |  |  |  |  |  |
|  | | | | | | | | | | | | | |
| *Note.*  M₁ includes gass_sex | | | | | | | | | | | | | |
| *Note.*  The intercept model is omitted, as no meaningful information can be shown. | | | | | | | | | | | | | |

| *Coefficients* | | | | | | | | | | | | | | | | | |
| --- | --- | --- | --- | --- | --- | --- | --- | --- | --- | --- | --- | --- | --- | --- | --- | --- | --- |
|  | | | | | | | | | | | | | | 95% CI | | | |
| Model | |  | | Unstandardized | | Standard Error | | Standardized | | t | | p | | Lower | | Upper | |
| M₀ |  | (Intercept) |  | 23.100 |  | 0.971 |  |  |  | 23.779 |  | < .001 |  | 21.172 |  | 25.028 |  |
| M₁ |  | (Intercept) |  | 21.980 |  | 1.135 |  |  |  | 19.372 |  | < .001 |  | 19.728 |  | 24.232 |  |
|  |  | gass_sex |  | 1.037 |  | 0.560 |  | 0.184 |  | 1.850 |  | 0.067 |  | -0.075 |  | 2.149 |  |
|  | | | | | | | | | | | | | | | | | |

**UKU total score**

| *Model Summary - whodas_total* | | | | | | | | | |
| --- | --- | --- | --- | --- | --- | --- | --- | --- | --- |
| Model | | R | | R² | | Adjusted R² | | RMSE | |
| M₀ |  | 0.000 |  | 0.000 |  | 0.000 |  | 9.715 |  |
| M₁ |  | 0.353 |  | 0.125 |  | 0.116 |  | 9.134 |  |
|  | | | | | | | | | |
| *Note.*  M₁ includes uku_tot | | | | | | | | | |

| *ANOVA* | | | | | | | | | | | | | |
| --- | --- | --- | --- | --- | --- | --- | --- | --- | --- | --- | --- | --- | --- |
| Model | |  | | Sum of Squares | | df | | Mean Square | | F | | p | |
| M₁ |  | Regression |  | 1166.840 |  | 1 |  | 1166.840 |  | 13.986 |  | < .001 |  |
|  |  | Residual |  | 8176.160 |  | 98 |  | 83.430 |  |  |  |  |  |
|  |  | Total |  | 9343.000 |  | 99 |  |  |  |  |  |  |  |
|  | | | | | | | | | | | | | |
| *Note.*  M₁ includes uku_tot | | | | | | | | | | | | | |
| *Note.*  The intercept model is omitted, as no meaningful information can be shown. | | | | | | | | | | | | | |

| *Coefficients* | | | | | | | | | | | | | | | | | |
| --- | --- | --- | --- | --- | --- | --- | --- | --- | --- | --- | --- | --- | --- | --- | --- | --- | --- |
|  | | | | | | | | | | | | | | 95% CI | | | |
| Model | |  | | Unstandardized | | Standard Error | | Standardized | | t | | p | | Lower | | Upper | |
| M₀ |  | (Intercept) |  | 23.100 |  | 0.971 |  |  |  | 23.779 |  | < .001 |  | 21.172 |  | 25.028 |  |
| M₁ |  | (Intercept) |  | 18.384 |  | 1.557 |  |  |  | 11.807 |  | < .001 |  | 15.294 |  | 21.474 |  |
|  |  | uku_tot |  | 0.667 |  | 0.178 |  | 0.353 |  | 3.740 |  | < .001 |  | 0.313 |  | 1.021 |  |
|  | | | | | | | | | | | | | | | | | |

**UKU psychological side effects**

| *Model Summary - whodas_total* | | | | | | | | | |
| --- | --- | --- | --- | --- | --- | --- | --- | --- | --- |
| Model | | R | | R² | | Adjusted R² | | RMSE | |
| M₀ |  | 0.000 |  | 0.000 |  | 0.000 |  | 9.715 |  |
| M₁ |  | 0.396 |  | 0.157 |  | 0.148 |  | 8.966 |  |
|  | | | | | | | | | |
| *Note.*  M₁ includes uku_psy | | | | | | | | | |

| *ANOVA* | | | | | | | | | | | | | |
| --- | --- | --- | --- | --- | --- | --- | --- | --- | --- | --- | --- | --- | --- |
| Model | |  | | Sum of Squares | | df | | Mean Square | | F | | p | |
| M₁ |  | Regression |  | 1464.938 |  | 1 |  | 1464.938 |  | 18.223 |  | < .001 |  |
|  |  | Residual |  | 7878.062 |  | 98 |  | 80.388 |  |  |  |  |  |
|  |  | Total |  | 9343.000 |  | 99 |  |  |  |  |  |  |  |
|  | | | | | | | | | | | | | |
| *Note.*  M₁ includes uku_psy | | | | | | | | | | | | | |
| *Note.*  The intercept model is omitted, as no meaningful information can be shown. | | | | | | | | | | | | | |

| *Coefficients* | | | | | | | | | | | | | | | | | |
| --- | --- | --- | --- | --- | --- | --- | --- | --- | --- | --- | --- | --- | --- | --- | --- | --- | --- |
|  | | | | | | | | | | | | | | 95% CI | | | |
| Model | |  | | Unstandardized | | Standard Error | | Standardized | | t | | p | | Lower | | Upper | |
| M₀ |  | (Intercept) |  | 23.100 |  | 0.971 |  |  |  | 23.779 |  | < .001 |  | 21.172 |  | 25.028 |  |
| M₁ |  | (Intercept) |  | 19.873 |  | 1.173 |  |  |  | 16.946 |  | < .001 |  | 17.546 |  | 22.200 |  |
|  |  | uku_psy |  | 3.752 |  | 0.879 |  | 0.396 |  | 4.269 |  | < .001 |  | 2.008 |  | 5.497 |  |
|  | | | | | | | | | | | | | | | | | |

**UKU neurological side effects**

| *Model Summary - whodas_total* | | | | | | | | | |
| --- | --- | --- | --- | --- | --- | --- | --- | --- | --- |
| Model | | R | | R² | | Adjusted R² | | RMSE | |
| M₀ |  | 0.000 |  | 0.000 |  | 0.000 |  | 9.715 |  |
| M₁ |  | 0.361 |  | 0.130 |  | 0.121 |  | 9.107 |  |
|  | | | | | | | | | |
| *Note.*  M₁ includes uku_neuro | | | | | | | | | |

| *ANOVA* | | | | | | | | | | | | | |
| --- | --- | --- | --- | --- | --- | --- | --- | --- | --- | --- | --- | --- | --- |
| Model | |  | | Sum of Squares | | df | | Mean Square | | F | | p | |
| M₁ |  | Regression |  | 1215.101 |  | 1 |  | 1215.101 |  | 14.651 |  | < .001 |  |
|  |  | Residual |  | 8127.899 |  | 98 |  | 82.938 |  |  |  |  |  |
|  |  | Total |  | 9343.000 |  | 99 |  |  |  |  |  |  |  |
|  | | | | | | | | | | | | | |
| *Note.*  M₁ includes uku_neuro | | | | | | | | | | | | | |
| *Note.*  The intercept model is omitted, as no meaningful information can be shown. | | | | | | | | | | | | | |

| *Coefficients* | | | | | | | | | | | | | | | | | |
| --- | --- | --- | --- | --- | --- | --- | --- | --- | --- | --- | --- | --- | --- | --- | --- | --- | --- |
|  | | | | | | | | | | | | | | 95% CI | | | |
| Model | |  | | Unstandardized | | Standard Error | | Standardized | | t | | p | | Lower | | Upper | |
| M₀ |  | (Intercept) |  | 23.100 |  | 0.971 |  |  |  | 23.779 |  | < .001 |  | 21.172 |  | 25.028 |  |
| M₁ |  | (Intercept) |  | 20.762 |  | 1.097 |  |  |  | 18.933 |  | < .001 |  | 18.586 |  | 22.938 |  |
|  |  | uku_neuro |  | 3.653 |  | 0.954 |  | 0.361 |  | 3.828 |  | < .001 |  | 1.759 |  | 5.547 |  |
|  | | | | | | | | | | | | | | | | | |

**UKU sexual side effects**

| *Model Summary - whodas_total* | | | | | | | | | |
| --- | --- | --- | --- | --- | --- | --- | --- | --- | --- |
| Model | | R | | R² | | Adjusted R² | | RMSE | |
| M₀ |  | 0.000 |  | 0.000 |  | 0.000 |  | 9.715 |  |
| M₁ |  | 0.152 |  | 0.023 |  | 0.013 |  | 9.650 |  |
|  | | | | | | | | | |
| *Note.*  M₁ includes uku_sex | | | | | | | | | |

| *ANOVA* | | | | | | | | | | | | | |
| --- | --- | --- | --- | --- | --- | --- | --- | --- | --- | --- | --- | --- | --- |
| Model | |  | | Sum of Squares | | df | | Mean Square | | F | | p | |
| M₁ |  | Regression |  | 217.122 |  | 1 |  | 217.122 |  | 2.332 |  | 0.130 |  |
|  |  | Residual |  | 9125.878 |  | 98 |  | 93.121 |  |  |  |  |  |
|  |  | Total |  | 9343.000 |  | 99 |  |  |  |  |  |  |  |
|  | | | | | | | | | | | | | |
| *Note.*  M₁ includes uku_sex | | | | | | | | | | | | | |
| *Note.*  The intercept model is omitted, as no meaningful information can be shown. | | | | | | | | | | | | | |

| *Coefficients* | | | | | | | | | | | | | | | | | |
| --- | --- | --- | --- | --- | --- | --- | --- | --- | --- | --- | --- | --- | --- | --- | --- | --- | --- |
|  | | | | | | | | | | | | | | 95% CI | | | |
| Model | |  | | Unstandardized | | Standard Error | | Standardized | | t | | p | | Lower | | Upper | |
| M₀ |  | (Intercept) |  | 23.100 |  | 0.971 |  |  |  | 23.779 |  | < .001 |  | 21.172 |  | 25.028 |  |
| M₁ |  | (Intercept) |  | 22.204 |  | 1.129 |  |  |  | 19.659 |  | < .001 |  | 19.963 |  | 24.445 |  |
|  |  | uku_sex |  | 0.586 |  | 0.384 |  | 0.152 |  | 1.527 |  | 0.130 |  | -0.175 |  | 1.347 |  |
|  | | | | | | | | | | | | | | | | | |

**UKU autonomic side effects**

| *Model Summary - whodas_total* | | | | | | | | | |
| --- | --- | --- | --- | --- | --- | --- | --- | --- | --- |
| Model | | R | | R² | | Adjusted R² | | RMSE | |
| M₀ |  | 0.000 |  | 0.000 |  | 0.000 |  | 9.715 |  |
| M₁ |  | 0.190 |  | 0.036 |  | 0.026 |  | 9.585 |  |
|  | | | | | | | | | |
| *Note.*  M₁ includes uku_auto | | | | | | | | | |

| *ANOVA* | | | | | | | | | | | | | |
| --- | --- | --- | --- | --- | --- | --- | --- | --- | --- | --- | --- | --- | --- |
| Model | |  | | Sum of Squares | | df | | Mean Square | | F | | p | |
| M₁ |  | Regression |  | 338.983 |  | 1 |  | 338.983 |  | 3.689 |  | 0.058 |  |
|  |  | Residual |  | 9004.017 |  | 98 |  | 91.878 |  |  |  |  |  |
|  |  | Total |  | 9343.000 |  | 99 |  |  |  |  |  |  |  |
|  | | | | | | | | | | | | | |
| *Note.*  M₁ includes uku_auto | | | | | | | | | | | | | |
| *Note.*  The intercept model is omitted, as no meaningful information can be shown. | | | | | | | | | | | | | |

| *Coefficients* | | | | | | | | | | | | | | | | | |
| --- | --- | --- | --- | --- | --- | --- | --- | --- | --- | --- | --- | --- | --- | --- | --- | --- | --- |
|  | | | | | | | | | | | | | | 95% CI | | | |
| Model | |  | | Unstandardized | | Standard Error | | Standardized | | t | | p | | Lower | | Upper | |
| M₀ |  | (Intercept) |  | 23.100 |  | 0.971 |  |  |  | 23.779 |  | < .001 |  | 21.172 |  | 25.028 |  |
| M₁ |  | (Intercept) |  | 20.870 |  | 1.506 |  |  |  | 13.862 |  | < .001 |  | 17.882 |  | 23.858 |  |
|  |  | uku_auto |  | 0.799 |  | 0.416 |  | 0.190 |  | 1.921 |  | 0.058 |  | -0.026 |  | 1.625 |  |
|  | | | | | | | | | | | | | | | | | |

**Linear regression analyses for quality of life (EQ-5D-5L)**

**GASS total score**

| *Model Summary - health_today* | | | | | | | | | |
| --- | --- | --- | --- | --- | --- | --- | --- | --- | --- |
| Model | | R | | R² | | Adjusted R² | | RMSE | |
| M₀ |  | 0.000 |  | 0.000 |  | 0.000 |  | 23.031 |  |
| M₁ |  | 0.398 |  | 0.158 |  | 0.150 |  | 21.238 |  |
|  | | | | | | | | | |
| *Note.*  M₁ includes gass_total | | | | | | | | | |

| *ANOVA* | | | | | | | | | | | | | |
| --- | --- | --- | --- | --- | --- | --- | --- | --- | --- | --- | --- | --- | --- |
| Model | |  | | Sum of Squares | | df | | Mean Square | | F | | p | |
| M₁ |  | Regression |  | 8307.608 |  | 1 |  | 8307.608 |  | 18.418 |  | < .001 |  |
|  |  | Residual |  | 44204.502 |  | 98 |  | 451.066 |  |  |  |  |  |
|  |  | Total |  | 52512.110 |  | 99 |  |  |  |  |  |  |  |
|  | | | | | | | | | | | | | |
| *Note.*  M₁ includes gass_total | | | | | | | | | | | | | |
| *Note.*  The intercept model is omitted, as no meaningful information can be shown. | | | | | | | | | | | | | |

| *Coefficients* | | | | | | | | | | | | | | | | | |
| --- | --- | --- | --- | --- | --- | --- | --- | --- | --- | --- | --- | --- | --- | --- | --- | --- | --- |
|  | | | | | | | | | | | | | | 95% CI | | | |
| Model | |  | | Unstandardized | | Standard Error | | Standardized | | t | | p | | Lower | | Upper | |
| M₀ |  | (Intercept) |  | 68.170 |  | 2.303 |  |  |  | 29.599 |  | < .001 |  | 63.600 |  | 72.740 |  |
| M₁ |  | (Intercept) |  | 81.718 |  | 3.805 |  |  |  | 21.478 |  | < .001 |  | 74.167 |  | 89.268 |  |
|  |  | gass_total |  | -0.951 |  | 0.222 |  | -0.398 |  | -4.292 |  | < .001 |  | -1.391 |  | -0.511 |  |
|  | | | | | | | | | | | | | | | | | |

**GASS psychological side effects**

| *Model Summary - health_today* | | | | | | | | | |
| --- | --- | --- | --- | --- | --- | --- | --- | --- | --- |
| Model | | R | | R² | | Adjusted R² | | RMSE | |
| M₀ |  | 0.000 |  | 0.000 |  | 0.000 |  | 23.031 |  |
| M₁ |  | 0.335 |  | 0.112 |  | 0.103 |  | 21.810 |  |
|  | | | | | | | | | |
| *Note.*  M₁ includes gass_psy | | | | | | | | | |

| *ANOVA* | | | | | | | | | | | | | |
| --- | --- | --- | --- | --- | --- | --- | --- | --- | --- | --- | --- | --- | --- |
| Model | |  | | Sum of Squares | | df | | Mean Square | | F | | p | |
| M₁ |  | Regression |  | 5894.785 |  | 1 |  | 5894.785 |  | 12.392 |  | < .001 |  |
|  |  | Residual |  | 46617.325 |  | 98 |  | 475.687 |  |  |  |  |  |
|  |  | Total |  | 52512.110 |  | 99 |  |  |  |  |  |  |  |
|  | | | | | | | | | | | | | |
| *Note.*  M₁ includes gass_psy | | | | | | | | | | | | | |
| *Note.*  The intercept model is omitted, as no meaningful information can be shown. | | | | | | | | | | | | | |

| *Coefficients* | | | | | | | | | | | | | | | | | |
| --- | --- | --- | --- | --- | --- | --- | --- | --- | --- | --- | --- | --- | --- | --- | --- | --- | --- |
|  | | | | | | | | | | | | | | 95% CI | | | |
| Model | |  | | Unstandardized | | Standard Error | | Standardized | | t | | p | | Lower | | Upper | |
| M₀ |  | (Intercept) |  | 68.170 |  | 2.303 |  |  |  | 29.599 |  | < .001 |  | 63.600 |  | 72.740 |  |
| M₁ |  | (Intercept) |  | 77.693 |  | 3.475 |  |  |  | 22.358 |  | < .001 |  | 70.797 |  | 84.589 |  |
|  |  | gass_psy |  | -4.762 |  | 1.353 |  | -0.335 |  | -3.520 |  | < .001 |  | -7.446 |  | -2.077 |  |
|  | | | | | | | | | | | | | | | | | |

**GASS neurological side effects**

| *Model Summary - health_today* | | | | | | | | | |
| --- | --- | --- | --- | --- | --- | --- | --- | --- | --- |
| Model | | R | | R² | | Adjusted R² | | RMSE | |
| M₀ |  | 0.000 |  | 0.000 |  | 0.000 |  | 23.031 |  |
| M₁ |  | 0.343 |  | 0.118 |  | 0.109 |  | 21.741 |  |
|  | | | | | | | | | |
| *Note.*  M₁ includes gass_neuro | | | | | | | | | |

| *ANOVA* | | | | | | | | | | | | | |
| --- | --- | --- | --- | --- | --- | --- | --- | --- | --- | --- | --- | --- | --- |
| Model | |  | | Sum of Squares | | df | | Mean Square | | F | | p | |
| M₁ |  | Regression |  | 6188.658 |  | 1 |  | 6188.658 |  | 13.092 |  | < .001 |  |
|  |  | Residual |  | 46323.452 |  | 98 |  | 472.688 |  |  |  |  |  |
|  |  | Total |  | 52512.110 |  | 99 |  |  |  |  |  |  |  |
|  | | | | | | | | | | | | | |
| *Note.*  M₁ includes gass_neuro | | | | | | | | | | | | | |
| *Note.*  The intercept model is omitted, as no meaningful information can be shown. | | | | | | | | | | | | | |

| *Coefficients* | | | | | | | | | | | | | | | | | |
| --- | --- | --- | --- | --- | --- | --- | --- | --- | --- | --- | --- | --- | --- | --- | --- | --- | --- |
|  | | | | | | | | | | | | | | 95% CI | | | |
| Model | |  | | Unstandardized | | Standard Error | | Standardized | | t | | p | | Lower | | Upper | |
| M₀ |  | (Intercept) |  | 68.170 |  | 2.303 |  |  |  | 29.599 |  | < .001 |  | 63.600 |  | 72.740 |  |
| M₁ |  | (Intercept) |  | 76.469 |  | 3.160 |  |  |  | 24.197 |  | < .001 |  | 70.197 |  | 82.740 |  |
|  |  | gass_neuro |  | -2.292 |  | 0.634 |  | -0.343 |  | -3.618 |  | < .001 |  | -3.550 |  | -1.035 |  |
|  | | | | | | | | | | | | | | | | | |

**GASS sexual side effects**

| *Model Summary - health_today* | | | | | | | | | |
| --- | --- | --- | --- | --- | --- | --- | --- | --- | --- |
| Model | | R | | R² | | Adjusted R² | | RMSE | |
| M₀ |  | 0.000 |  | 0.000 |  | 0.000 |  | 23.031 |  |
| M₁ |  | 0.127 |  | 0.016 |  | 0.006 |  | 22.961 |  |
|  | | | | | | | | | |
| *Note.*  M₁ includes gass_sex | | | | | | | | | |

| *ANOVA* | | | | | | | | | | | | | |
| --- | --- | --- | --- | --- | --- | --- | --- | --- | --- | --- | --- | --- | --- |
| Model | |  | | Sum of Squares | | df | | Mean Square | | F | | p | |
| M₁ |  | Regression |  | 846.614 |  | 1 |  | 846.614 |  | 1.606 |  | 0.208 |  |
|  |  | Residual |  | 51665.496 |  | 98 |  | 527.199 |  |  |  |  |  |
|  |  | Total |  | 52512.110 |  | 99 |  |  |  |  |  |  |  |
|  | | | | | | | | | | | | | |
| *Note.*  M₁ includes gass_sex | | | | | | | | | | | | | |
| *Note.*  The intercept model is omitted, as no meaningful information can be shown. | | | | | | | | | | | | | |

| *Coefficients* | | | | | | | | | | | | | | | | | |
| --- | --- | --- | --- | --- | --- | --- | --- | --- | --- | --- | --- | --- | --- | --- | --- | --- | --- |
|  | | | | | | | | | | | | | | 95% CI | | | |
| Model | |  | | Unstandardized | | Standard Error | | Standardized | | t | | p | | Lower | | Upper | |
| M₀ |  | (Intercept) |  | 68.170 |  | 2.303 |  |  |  | 29.599 |  | < .001 |  | 63.600 |  | 72.740 |  |
| M₁ |  | (Intercept) |  | 70.005 |  | 2.714 |  |  |  | 25.790 |  | < .001 |  | 64.618 |  | 75.391 |  |
|  |  | gass_sex |  | -1.699 |  | 1.341 |  | -0.127 |  | -1.267 |  | 0.208 |  | -4.359 |  | 0.962 |  |
|  | | | | | | | | | | | | | | | | | |

**GASS autonomic side effects**

| *Model Summary - health_today* | | | | | | | | | |
| --- | --- | --- | --- | --- | --- | --- | --- | --- | --- |
| Model | | R | | R² | | Adjusted R² | | RMSE | |
| M₀ |  | 0.000 |  | 0.000 |  | 0.000 |  | 23.031 |  |
| M₁ |  | 0.369 |  | 0.136 |  | 0.127 |  | 21.514 |  |
|  | | | | | | | | | |
| *Note.*  M₁ includes gass_auto | | | | | | | | | |

| *ANOVA* | | | | | | | | | | | | | |
| --- | --- | --- | --- | --- | --- | --- | --- | --- | --- | --- | --- | --- | --- |
| Model | |  | | Sum of Squares | | df | | Mean Square | | F | | p | |
| M₁ |  | Regression |  | 7154.295 |  | 1 |  | 7154.295 |  | 15.458 |  | < .001 |  |
|  |  | Residual |  | 45357.815 |  | 98 |  | 462.835 |  |  |  |  |  |
|  |  | Total |  | 52512.110 |  | 99 |  |  |  |  |  |  |  |
|  | | | | | | | | | | | | | |
| *Note.*  M₁ includes gass_auto | | | | | | | | | | | | | |
| *Note.*  The intercept model is omitted, as no meaningful information can be shown. | | | | | | | | | | | | | |

| *Coefficients* | | | | | | | | | | | | | | | | | |
| --- | --- | --- | --- | --- | --- | --- | --- | --- | --- | --- | --- | --- | --- | --- | --- | --- | --- |
|  | | | | | | | | | | | | | | 95% CI | | | |
| Model | |  | | Unstandardized | | Standard Error | | Standardized | | t | | p | | Lower | | Upper | |
| M₀ |  | (Intercept) |  | 68.170 |  | 2.303 |  |  |  | 29.599 |  | < .001 |  | 63.600 |  | 72.740 |  |
| M₁ |  | (Intercept) |  | 78.858 |  | 3.467 |  |  |  | 22.747 |  | < .001 |  | 71.979 |  | 85.738 |  |
|  |  | gass_auto |  | -1.799 |  | 0.458 |  | -0.369 |  | -3.932 |  | < .001 |  | -2.708 |  | -0.891 |  |
|  | | | | | | | | | | | | | | | | | |

**UKU total score**

| *Model Summary - health_today* | | | | | | | | | |
| --- | --- | --- | --- | --- | --- | --- | --- | --- | --- |
| Model | | R | | R² | | Adjusted R² | | RMSE | |
| M₀ |  | 0.000 |  | 0.000 |  | 0.000 |  | 23.031 |  |
| M₁ |  | 0.541 |  | 0.293 |  | 0.286 |  | 19.464 |  |
|  | | | | | | | | | |
| *Note.*  M₁ includes uku_tot | | | | | | | | | |

| *ANOVA* | | | | | | | | | | | | | |
| --- | --- | --- | --- | --- | --- | --- | --- | --- | --- | --- | --- | --- | --- |
| Model | |  | | Sum of Squares | | df | | Mean Square | | F | | p | |
| M₁ |  | Regression |  | 15386.144 |  | 1 |  | 15386.144 |  | 40.614 |  | < .001 |  |
|  |  | Residual |  | 37125.966 |  | 98 |  | 378.836 |  |  |  |  |  |
|  |  | Total |  | 52512.110 |  | 99 |  |  |  |  |  |  |  |
|  | | | | | | | | | | | | | |
| *Note.*  M₁ includes uku_tot | | | | | | | | | | | | | |
| *Note.*  The intercept model is omitted, as no meaningful information can be shown. | | | | | | | | | | | | | |

| *Coefficients* | | | | | | | | | | | | | | | | | |
| --- | --- | --- | --- | --- | --- | --- | --- | --- | --- | --- | --- | --- | --- | --- | --- | --- | --- |
|  | | | | | | | | | | | | | | 95% CI | | | |
| Model | |  | | Unstandardized | | Standard Error | | Standardized | | t | | p | | Lower | | Upper | |
| M₀ |  | (Intercept) |  | 68.170 |  | 2.303 |  |  |  | 29.599 |  | < .001 |  | 63.600 |  | 72.740 |  |
| M₁ |  | (Intercept) |  | 85.295 |  | 3.318 |  |  |  | 25.707 |  | < .001 |  | 78.710 |  | 91.879 |  |
|  |  | uku_tot |  | -2.422 |  | 0.380 |  | -0.541 |  | -6.373 |  | < .001 |  | -3.176 |  | -1.668 |  |
|  | | | | | | | | | | | | | | | | | |

**UKU psychological side effects**

| *Model Summary - health_today* | | | | | | | | | |
| --- | --- | --- | --- | --- | --- | --- | --- | --- | --- |
| Model | | R | | R² | | Adjusted R² | | RMSE | |
| M₀ |  | 0.000 |  | 0.000 |  | 0.000 |  | 23.031 |  |
| M₁ |  | 0.455 |  | 0.207 |  | 0.199 |  | 20.618 |  |
|  | | | | | | | | | |
| *Note.*  M₁ includes uku_psy | | | | | | | | | |

| *ANOVA* | | | | | | | | | | | | | |
| --- | --- | --- | --- | --- | --- | --- | --- | --- | --- | --- | --- | --- | --- |
| Model | |  | | Sum of Squares | | df | | Mean Square | | F | | p | |
| M₁ |  | Regression |  | 10853.146 |  | 1 |  | 10853.146 |  | 25.531 |  | < .001 |  |
|  |  | Residual |  | 41658.964 |  | 98 |  | 425.091 |  |  |  |  |  |
|  |  | Total |  | 52512.110 |  | 99 |  |  |  |  |  |  |  |
|  | | | | | | | | | | | | | |
| *Note.*  M₁ includes uku_psy | | | | | | | | | | | | | |
| *Note.*  The intercept model is omitted, as no meaningful information can be shown. | | | | | | | | | | | | | |

| *Coefficients* | | | | | | | | | | | | | | | | | |
| --- | --- | --- | --- | --- | --- | --- | --- | --- | --- | --- | --- | --- | --- | --- | --- | --- | --- |
|  | | | | | | | | | | | | | | 95% CI | | | |
| Model | |  | | Unstandardized | | Standard Error | | Standardized | | t | | p | | Lower | | Upper | |
| M₀ |  | (Intercept) |  | 68.170 |  | 2.303 |  |  |  | 29.599 |  | < .001 |  | 63.600 |  | 72.740 |  |
| M₁ |  | (Intercept) |  | 76.954 |  | 2.697 |  |  |  | 28.535 |  | < .001 |  | 71.602 |  | 82.305 |  |
|  |  | uku_psy |  | -10.214 |  | 2.021 |  | -0.455 |  | -5.053 |  | < .001 |  | -14.225 |  | -6.202 |  |
|  | | | | | | | | | | | | | | | | | |

**UKU neurological side effects**

| *Model Summary - health_today* | | | | | | | | | |
| --- | --- | --- | --- | --- | --- | --- | --- | --- | --- |
| Model | | R | | R² | | Adjusted R² | | RMSE | |
| M₀ |  | 0.000 |  | 0.000 |  | 0.000 |  | 23.031 |  |
| M₁ |  | 0.455 |  | 0.207 |  | 0.199 |  | 20.618 |  |
|  | | | | | | | | | |
| *Note.*  M₁ includes uku_psy | | | | | | | | | |

| *ANOVA* | | | | | | | | | | | | | |
| --- | --- | --- | --- | --- | --- | --- | --- | --- | --- | --- | --- | --- | --- |
| Model | |  | | Sum of Squares | | df | | Mean Square | | F | | p | |
| M₁ |  | Regression |  | 10853.146 |  | 1 |  | 10853.146 |  | 25.531 |  | < .001 |  |
|  |  | Residual |  | 41658.964 |  | 98 |  | 425.091 |  |  |  |  |  |
|  |  | Total |  | 52512.110 |  | 99 |  |  |  |  |  |  |  |
|  | | | | | | | | | | | | | |
| *Note.*  M₁ includes uku_psy | | | | | | | | | | | | | |
| *Note.*  The intercept model is omitted, as no meaningful information can be shown. | | | | | | | | | | | | | |

| *Coefficients* | | | | | | | | | | | | | | | | | |
| --- | --- | --- | --- | --- | --- | --- | --- | --- | --- | --- | --- | --- | --- | --- | --- | --- | --- |
|  | | | | | | | | | | | | | | 95% CI | | | |
| Model | |  | | Unstandardized | | Standard Error | | Standardized | | t | | p | | Lower | | Upper | |
| M₀ |  | (Intercept) |  | 68.170 |  | 2.303 |  |  |  | 29.599 |  | < .001 |  | 63.600 |  | 72.740 |  |
| M₁ |  | (Intercept) |  | 76.954 |  | 2.697 |  |  |  | 28.535 |  | < .001 |  | 71.602 |  | 82.305 |  |
|  |  | uku_psy |  | -10.214 |  | 2.021 |  | -0.455 |  | -5.053 |  | < .001 |  | -14.225 |  | -6.202 |  |
|  | | | | | | | | | | | | | | | | | |

**UKU sexual side effects**

| *Model Summary - health_today* | | | | | | | | | |
| --- | --- | --- | --- | --- | --- | --- | --- | --- | --- |
| Model | | R | | R² | | Adjusted R² | | RMSE | |
| M₀ |  | 0.000 |  | 0.000 |  | 0.000 |  | 23.031 |  |
| M₁ |  | 0.288 |  | 0.083 |  | 0.074 |  | 22.165 |  |
|  | | | | | | | | | |
| *Note.*  M₁ includes uku_sex | | | | | | | | | |

| *ANOVA* | | | | | | | | | | | | | |
| --- | --- | --- | --- | --- | --- | --- | --- | --- | --- | --- | --- | --- | --- |
| Model | |  | | Sum of Squares | | df | | Mean Square | | F | | p | |
| M₁ |  | Regression |  | 4364.408 |  | 1 |  | 4364.408 |  | 8.883 |  | 0.004 |  |
|  |  | Residual |  | 48147.702 |  | 98 |  | 491.303 |  |  |  |  |  |
|  |  | Total |  | 52512.110 |  | 99 |  |  |  |  |  |  |  |
|  | | | | | | | | | | | | | |
| *Note.*  M₁ includes uku_sex | | | | | | | | | | | | | |
| *Note.*  The intercept model is omitted, as no meaningful information can be shown. | | | | | | | | | | | | | |

| *Coefficients* | | | | | | | | | | | | | | | | | |
| --- | --- | --- | --- | --- | --- | --- | --- | --- | --- | --- | --- | --- | --- | --- | --- | --- | --- |
|  | | | | | | | | | | | | | | 95% CI | | | |
| Model | |  | | Unstandardized | | Standard Error | | Standardized | | t | | p | | Lower | | Upper | |
| M₀ |  | (Intercept) |  | 68.170 |  | 2.303 |  |  |  | 29.599 |  | < .001 |  | 63.600 |  | 72.740 |  |
| M₁ |  | (Intercept) |  | 72.188 |  | 2.594 |  |  |  | 27.826 |  | < .001 |  | 67.040 |  | 77.336 |  |
|  |  | uku_sex |  | -2.626 |  | 0.881 |  | -0.288 |  | -2.980 |  | 0.004 |  | -4.374 |  | -0.878 |  |
|  | | | | | | | | | | | | | | | | | |

**UKU autonomic side effects**

| *Model Summary - health_today* | | | | | | | | | |
| --- | --- | --- | --- | --- | --- | --- | --- | --- | --- |
| Model | | R | | R² | | Adjusted R² | | RMSE | |
| M₀ |  | 0.000 |  | 0.000 |  | 0.000 |  | 23.031 |  |
| M₁ |  | 0.378 |  | 0.143 |  | 0.134 |  | 21.429 |  |
|  | | | | | | | | | |
| *Note.*  M₁ includes uku_auto | | | | | | | | | |

| *ANOVA* | | | | | | | | | | | | | |
| --- | --- | --- | --- | --- | --- | --- | --- | --- | --- | --- | --- | --- | --- |
| Model | |  | | Sum of Squares | | df | | Mean Square | | F | | p | |
| M₁ |  | Regression |  | 7511.888 |  | 1 |  | 7511.888 |  | 16.359 |  | < .001 |  |
|  |  | Residual |  | 45000.222 |  | 98 |  | 459.186 |  |  |  |  |  |
|  |  | Total |  | 52512.110 |  | 99 |  |  |  |  |  |  |  |
|  | | | | | | | | | | | | | |
| *Note.*  M₁ includes uku_auto | | | | | | | | | | | | | |
| *Note.*  The intercept model is omitted, as no meaningful information can be shown. | | | | | | | | | | | | | |

| *Coefficients* | | | | | | | | | | | | | | | | | |
| --- | --- | --- | --- | --- | --- | --- | --- | --- | --- | --- | --- | --- | --- | --- | --- | --- | --- |
|  | | | | | | | | | | | | | | 95% CI | | | |
| Model | |  | | Unstandardized | | Standard Error | | Standardized | | t | | p | | Lower | | Upper | |
| M₀ |  | (Intercept) |  | 68.170 |  | 2.303 |  |  |  | 29.599 |  | < .001 |  | 63.600 |  | 72.740 |  |
| M₁ |  | (Intercept) |  | 78.668 |  | 3.366 |  |  |  | 23.373 |  | < .001 |  | 71.989 |  | 85.347 |  |
|  |  | uku_auto |  | -3.763 |  | 0.930 |  | -0.378 |  | -4.045 |  | < .001 |  | -5.609 |  | -1.917 |  |
|  | | | | | | | | | | | | | | | | | |

**Appendix C**

**Network analysis**

A separate analysis was conducted, focusing exclusively on antipsychotic side effects and symptoms, to better understand their direct impact on clinical outcomes and to identify specific symptom-side effect interactions that may be obscured in broader analyses. Single items from YMRS, MADRS, and GASS were analysed. Four distinct networks were generated, each representing a specific group of side effects (Figure S1). Low-density networks emerged, with reported sadness being the node with the greatest strength and expected influence in each one (Table S3).

**Fig. S1** Networks representing the interactions between symptoms and specific side effects

*mrs_1: elevated mood; mrs_2: increased motor activity-energy; mrs_3: sexual interest; mrs_4: sleep; mrs_5: irritability; mrs_6: speech; mrs_7: language-thought disorder; mrs_8: content; mrs_9: disruptive-aggressive behavior; mrs_10: appearance; mrs_11: insight; madrs_1: apparent sadness; madrs_2: reported sadness; madrs_3: inner tension; madrs_4: reduced sleep; madrs_5: reduced appetite; madrs_6: concentration difficulties; madrs_7: lassitude; madrs_8: inability to feel; madrs_9: pessimistic thoughts; madrs_10: suicidal thoughts; gass_01_freq_cat: asthenia; gass_02_freq_cat: sedation; gass_03_freq_cat: orthostatic hypotension; gass_04_freq_cat: palpitations; gass_05_freq_cat: dystonia; gass_06_freq_cat: tremor; gass_07_freq_cat: akathisia; gass_08_freq_cat: increased salivation; gass_09_freq_cat: hypokinesia; gass_10_freq_cat: hyperkinesia; gass_11_freq_cat: accomodation disturbances; gass_12_freq_cat: dry mouth; gass_13_freq_cat: dysuria; gass_14_freq_cat: nausea/vomiting; gass_15_freq_cat: nocturnal enuresis; gass_16_freq_cat: polyuria/polydipsia; gass_19_freq_cat: sexual dysfunction; gass_20_freq_cat: erectile dysfunction.*

**Table S3.** Nodes with the highest values in centrality measures and expected influence for each network

| *Network* | **Betweenness** | **Closeness** | **Strength** | **Expected influence** |
| --- | --- | --- | --- | --- |
| Autonomic SE | Dry mouth | Inability to feel | Reported sadness | Reported sadness |
| Neurological SE | Irritability | Lassitude | Reported sadness | Reported sadness |
| Psychological SE | Lassitude | Lassitude | Reported sadness | Reported sadness |
| Sexual SE | Sleep | Inability to feel | Reported sadness | Reported sadness |

This analysis examined each symptom and side effect individually, resulting in four distinct networks based on side effect subgroups. Reported sadness emerged as the most central and influential node across all networks, suggesting that it plays a pivotal role in how side effects interact with symptoms. Considering results across different networks, it can be noted that dry mouth (autonomic network), irritability (neurological network), lassitude (psychological network), and sleep disturbance (sexual network) act as key connecting nodes, facilitating interactions between other symptoms. Closeness highlights emotional symptoms such as lassitude and inability to feel emotions, indicating their strong association with multiple side effects. Strength and expected influence consistently point to reported sadness as a primary factor, reinforcing its role as a core symptom in all networks. These results suggest that reported sadness is a key symptom linked to various antipsychotic side effects, regardless of their category. Emotional symptoms are also highly central, emphasizing the importance of monitoring mood-related effects when assessing antipsychotic tolerability.

**Table S4.** Psychological side effects network

| *Summary of Network* | | | | | | | |  |  |  |
| --- | --- | --- | --- | --- | --- | --- | --- | --- | --- | --- |
| Number of nodes | | Number of non-zero edges | | | Sparsity | | |  |  |  |
| 23 |  | 53 / 253 | |  | 0.791 | |  |  |  |  |
|  | | | | | | | |  |  |  |
| Centrality measures per variable | | | | | | | |  |  |  |
|  | | Network | | | | | | | | |
| Variable | | Betweenness | | Closeness | | Strength | | | Expected influence | |
| gass_01_freq_cat |  | -0.994 |  | -0.268 |  | -1.480 | |  | -1.480 |  |
| gass_02_freq_cat |  | -0.994 |  | -0.843 |  | -1.505 | |  | -1.505 |  |
| madrs_1 |  | 0.234 |  | 0.848 |  | 1.463 | |  | 1.463 |  |
| madrs_2 |  | 0.572 |  | 0.933 |  | 2.127 | |  | 2.127 |  |
| madrs_3 |  | 0.022 |  | 0.780 |  | 0.423 | |  | 0.423 |  |
| madrs_4 |  | 0.361 |  | 0.706 |  | 0.792 | |  | 0.792 |  |
| madrs_5 |  | -0.994 |  | 0.185 |  | 0.040 | |  | 0.040 |  |
| madrs_6 |  | -0.486 |  | 0.666 |  | -0.321 | |  | -0.321 |  |
| madrs_7 |  | 1.885 |  | 1.120 |  | 1.782 | |  | 1.782 |  |
| madrs_8 |  | 1.546 |  | 1.105 |  | 0.339 | |  | 0.339 |  |
| madrs_9 |  | -0.825 |  | 0.170 |  | -0.468 | |  | -0.468 |  |
| madrs_10 |  | -0.994 |  | -0.111 |  | -0.450 | |  | -0.450 |  |
| mrs_1 |  | -0.994 |  | -2.111 |  | -0.850 | |  | -0.850 |  |
| mrs_2 |  | -0.063 |  | -2.052 |  | -0.058 | |  | -0.058 |  |
| mrs_3 |  | -0.994 |  | -0.278 |  | -0.350 | |  | -0.350 |  |
| mrs_4 |  | 1.546 |  | 0.735 |  | 0.844 | |  | 0.844 |  |
| mrs_5 |  | -0.063 |  | 0.576 |  | 0.051 | |  | 0.051 |  |
| mrs_6 |  | 0.022 |  | 0.227 |  | -0.213 | |  | -0.213 |  |
| mrs_7 |  | 0.742 |  | 0.158 |  | 0.924 | |  | 0.924 |  |
| mrs_8 |  | -0.994 |  | -0.449 |  | -0.516 | |  | -0.516 |  |
| mrs_9 |  | 1.843 |  | 0.853 |  | -0.079 | |  | -0.079 |  |
| mrs_10 |  | 0.615 |  | -2.001 |  | -0.795 | |  | -0.795 |  |
| mrs_11 |  | -0.994 |  | -0.950 |  | -1.701 | |  | -1.701 |  |
|  | | | | | | | | | | |

**Table S5.** Neurological side effects network

| *Summary of Network* | | | | | | | |  |  |  |
| --- | --- | --- | --- | --- | --- | --- | --- | --- | --- | --- |
| Number of nodes | | Number of non-zero edges | | | Sparsity | | |  |  |  |
| 26 |  | 57 / 325 | |  | 0.825 | |  |  |  |  |
|  | | | | | | | |  |  |  |
| Centrality measures per variable | | | | | | | |  |  |  |
|  | | Network | | | | | | | | |
| Variable | | Betweenness | | Closeness | | Strength | | | Expected influence | |
| gass_05_freq_cat |  | -0.965 |  | 0.000 |  | -1.282 | |  | -1.261 |  |
| gass_06_freq_cat |  | 0.489 |  | 0.000 |  | -0.043 | |  | -0.150 |  |
| gass_07_freq_cat |  | -0.104 |  | 0.000 |  | -0.492 | |  | -0.478 |  |
| gass_09_freq_cat |  | 1.296 |  | 0.000 |  | -0.025 | |  | -0.016 |  |
| gass_10_freq_cat |  | -0.588 |  | 0.000 |  | -0.531 | |  | -0.517 |  |
| madrs_1 |  | 0.650 |  | 0.000 |  | 1.699 | |  | 1.691 |  |
| madrs_2 |  | 0.542 |  | 0.000 |  | 2.477 | |  | 2.462 |  |
| madrs_3 |  | 1.135 |  | 0.000 |  | 0.371 | |  | 0.376 |  |
| madrs_4 |  | -0.265 |  | 0.000 |  | 0.893 | |  | 0.894 |  |
| madrs_5 |  | -0.965 |  | 0.000 |  | -0.004 | |  | 0.005 |  |
| madrs_6 |  | -0.965 |  | 0.000 |  | -0.491 | |  | -0.477 |  |
| madrs_7 |  | 1.350 |  | 0.000 |  | 1.926 | |  | 1.916 |  |
| madrs_8 |  | 0.489 |  | 0.000 |  | 0.268 | |  | 0.275 |  |
| madrs_9 |  | -0.319 |  | 0.000 |  | -0.579 | |  | -0.565 |  |
| madrs_10 |  | 0.273 |  | 0.000 |  | -0.076 | |  | -0.066 |  |
| mrs_1 |  | -0.965 |  | 0.000 |  | -1.015 | |  | -0.996 |  |
| mrs_2 |  | -0.911 |  | 0.000 |  | -0.173 | |  | -0.163 |  |
| mrs_3 |  | -0.965 |  | 0.000 |  | -0.432 | |  | -0.419 |  |
| mrs_4 |  | -0.750 |  | 0.000 |  | 0.881 | |  | 0.882 |  |
| mrs_5 |  | 2.696 |  | 0.000 |  | 0.293 | |  | 0.299 |  |
| mrs_6 |  | -0.319 |  | 0.000 |  | -0.328 | |  | -0.316 |  |
| mrs_7 |  | 1.242 |  | 0.000 |  | 0.849 | |  | 0.850 |  |
| mrs_8 |  | -0.965 |  | 0.000 |  | -0.680 | |  | -0.665 |  |
| mrs_9 |  | 0.812 |  | 0.000 |  | -0.552 | |  | -0.537 |  |
| mrs_10 |  | -0.965 |  | 0.000 |  | -1.012 | |  | -0.993 |  |
| mrs_11 |  | -0.965 |  | 0.000 |  | -1.944 | |  | -2.032 |  |
|  | | | | | | | | | | |

**Table S6.** Autonomic side effects network

| *Summary of Network* | | | | | | | |  |  |  |
| --- | --- | --- | --- | --- | --- | --- | --- | --- | --- | --- |
| Number of nodes | | Number of non-zero edges | | | Sparsity | | |  |  |  |
| 30 |  | 84 / 435 | |  | 0.807 | |  |  |  |  |
|  | | | | | | | |  |  |  |
| Centrality measures per variable | | | | | | | |  |  |  |
|  | | Network | | | | | | | | |
| Variable | | Betweenness | | Closeness | | Strength | | | Expected influence | |
| gass_04_freq_cat |  | -0.850 |  | 0.024 |  | -0.900 | |  | -0.900 |  |
| gass_08_freq_cat |  | -0.610 |  | 0.211 |  | -0.567 | |  | -0.567 |  |
| gass_11_freq_cat |  | 0.315 |  | 0.412 |  | 0.685 | |  | 0.685 |  |
| gass_03_freq_cat |  | -0.233 |  | 0.185 |  | -0.208 | |  | -0.208 |  |
| gass_12_freq_cat |  | 3.501 |  | 1.074 |  | 0.900 | |  | 0.900 |  |
| gass_13_freq_cat |  | -0.747 |  | -0.064 |  | -0.980 | |  | -0.980 |  |
| gass_14_freq_cat |  | -1.055 |  | -0.468 |  | -1.132 | |  | -1.132 |  |
| gass_15_freq_cat |  | -1.055 |  | -1.666 |  | -1.806 | |  | -1.806 |  |
| gass_16_freq_cat |  | 1.549 |  | 0.083 |  | -1.335 | |  | -1.335 |  |
| mrs_1 |  | 0.726 |  | -2.174 |  | -0.819 | |  | -0.819 |  |
| mrs_2 |  | -0.164 |  | -2.298 |  | -0.092 | |  | -0.092 |  |
| mrs_3 |  | -0.644 |  | 0.263 |  | -0.033 | |  | -0.033 |  |
| mrs_4 |  | -0.473 |  | 0.303 |  | 1.041 | |  | 1.041 |  |
| mrs_5 |  | -0.096 |  | 0.516 |  | 0.056 | |  | 0.056 |  |
| mrs_6 |  | -0.644 |  | 0.242 |  | -0.254 | |  | -0.254 |  |
| mrs_7 |  | 0.692 |  | 0.535 |  | 0.924 | |  | 0.924 |  |
| mrs_8 |  | 0.075 |  | 0.306 |  | -0.188 | |  | -0.188 |  |
| mrs_9 |  | 0.349 |  | 0.587 |  | -0.225 | |  | -0.225 |  |
| mrs_10 |  | -1.055 |  | -2.409 |  | -0.866 | |  | -0.866 |  |
| mrs_11 |  | -1.055 |  | -1.599 |  | -1.817 | |  | -1.817 |  |
| madrs_1 |  | 0.247 |  | 0.869 |  | 1.730 | |  | 1.730 |  |
| madrs_2 |  | 0.212 |  | 0.901 |  | 2.320 | |  | 2.320 |  |
| madrs_3 |  | 0.041 |  | 0.579 |  | 0.705 | |  | 0.705 |  |
| madrs_4 |  | -0.610 |  | 0.282 |  | 0.864 | |  | 0.864 |  |
| madrs_5 |  | -1.055 |  | -0.038 |  | 0.245 | |  | 0.245 |  |
| madrs_6 |  | -0.404 |  | 0.754 |  | -0.093 | |  | -0.093 |  |
| madrs_7 |  | 0.863 |  | 0.901 |  | 1.530 | |  | 1.530 |  |
| madrs_8 |  | 1.754 |  | 1.126 |  | 0.554 | |  | 0.554 |  |
| madrs_9 |  | 0.521 |  | 0.613 |  | 0.075 | |  | 0.075 |  |
| madrs_10 |  | -0.096 |  | -0.049 |  | -0.315 | |  | -0.315 |  |
|  | | | | | | | | | | |

**Table S7.** Sexual side effects network

| *Summary of Network* | | | | | | | |  |  |  |
| --- | --- | --- | --- | --- | --- | --- | --- | --- | --- | --- |
| Number of nodes | | Number of non-zero edges | | | Sparsity | | |  |  |  |
| 23 |  | 51 / 253 | |  | 0.798 | |  |  |  |  |
|  | | | | | | | |  |  |  |
| Centrality measures per variable | | | | | | | |  |  |  |
|  | | Network | | | | | | | | |
| Variable | | Betweenness | | Closeness | | Strength | | | Expected influence | |
| gass_10_freq_cat |  | -1.129 |  | -1.747 |  | -1.827 | |  | -1.827 |  |
| gass_20_freq_cat |  | -1.129 |  | -0.734 |  | -1.508 | |  | -1.508 |  |
| madrs_1 |  | 0.215 |  | 0.799 |  | 1.451 | |  | 1.451 |  |
| madrs_10 |  | -1.129 |  | -0.212 |  | -0.435 | |  | -0.435 |  |
| madrs_2 |  | -0.002 |  | 0.821 |  | 2.110 | |  | 2.110 |  |
| madrs_3 |  | 0.215 |  | 0.866 |  | 0.458 | |  | 0.458 |  |
| madrs_4 |  | 0.085 |  | 0.589 |  | 0.796 | |  | 0.796 |  |
| madrs_5 |  | -1.129 |  | 0.137 |  | 0.041 | |  | 0.041 |  |
| madrs_6 |  | -0.479 |  | 0.716 |  | -0.356 | |  | -0.356 |  |
| madrs_7 |  | 1.255 |  | 1.053 |  | 1.483 | |  | 1.483 |  |
| madrs_8 |  | 1.168 |  | 1.056 |  | 0.362 | |  | 0.362 |  |
| madrs_9 |  | -0.955 |  | 0.087 |  | -0.466 | |  | -0.466 |  |
| mrs_1 |  | -1.129 |  | -1.959 |  | -0.844 | |  | -0.844 |  |
| mrs_10 |  | 0.518 |  | -1.795 |  | -0.751 | |  | -0.751 |  |
| mrs_11 |  | -1.129 |  | -0.680 |  | -1.673 | |  | -1.673 |  |
| mrs_2 |  | -0.175 |  | -1.872 |  | -0.016 | |  | -0.016 |  |
| mrs_3 |  | 0.648 |  | -0.009 |  | 0.186 | |  | 0.186 |  |
| mrs_4 |  | 1.428 |  | 0.619 |  | 0.865 | |  | 0.865 |  |
| mrs_5 |  | 0.215 |  | 0.742 |  | 0.079 | |  | 0.079 |  |
| mrs_6 |  | 0.388 |  | 0.438 |  | -0.198 | |  | -0.198 |  |
| mrs_7 |  | 2.035 |  | 0.434 |  | 0.958 | |  | 0.958 |  |
| mrs_8 |  | -1.129 |  | -0.241 |  | -0.504 | |  | -0.504 |  |
| mrs_9 |  | 1.342 |  | 0.891 |  | -0.212 | |  | -0.212 |  |
|  | | | | | | | | | | |

**Table S8.** Interactions between disability, quality of life, symptoms and side effects using GASS

| *Summary of Network* | | | | | | | | | | | |  |
| --- | --- | --- | --- | --- | --- | --- | --- | --- | --- | --- | --- | --- |
| Number of nodes | | | Number of non-zero edges | | | | | Sparsity | | | |  |
| 8 |  | | 16 / 28 | | |  | | 0.429 | |  | |  |
|  | | | | | | | | | | | |  |
| Centrality measures per variable | | | | | | | | | | | |  |
|  | | Network | | | | | | | | | | |
| Variable | | Betweenness | | | Closeness | | | Strength | | | Expected influence | |
| gass_auto |  | 2.262 | |  | 1.216 | |  | 1.526 |  | | 1.371 |  |
| gass_neuro |  | -0.036 | |  | 1.052 | |  | 0.549 |  | | 0.967 |  |
| gass_psy |  | -0.610 | |  | -0.885 | |  | -0.489 |  | | -0.003 |  |
| gass_sex |  | -0.610 | |  | -0.516 | |  | -1.129 |  | | -0.039 |  |
| health_today |  | -0.610 | |  | -0.226 | |  | 0.064 |  | | -2.024 |  |
| madrs_tot |  | 0.538 | |  | 0.537 | |  | 0.461 |  | | 0.018 |  |
| mrs_tot |  | -0.610 | |  | -1.669 | |  | -1.530 |  | | -0.297 |  |
| whodas_total |  | -0.323 | |  | 0.491 | |  | 0.548 |  | | 0.008 |  |
|  | | | | | | | | | | | | |

**Table S9.** Interactions between disability, quality of life, symptoms and side effects using UKU

| *Summary of Network* | | | | | | | | | | | |  |
| --- | --- | --- | --- | --- | --- | --- | --- | --- | --- | --- | --- | --- |
| Number of nodes | | | Number of non-zero edges | | | | | Sparsity | | | |  |
| 8 |  | | 17 / 28 | | |  | | 0.393 | |  | |  |
|  | | | | | | | | | | | |  |
| Centrality measures per variable | | | | | | | | | | | |  |
|  | | Network | | | | | | | | | | |
| Variable | | Betweenness | | | Closeness | | | Strength | | | Expected influence | |
| health_today |  | 0.262 | |  | 0.889 | |  | 1.193 |  | | -2.227 |  |
| madrs_tot |  | 2.358 | |  | 1.327 | |  | 1.351 |  | | 1.246 |  |
| mrs_tot |  | -0.524 | |  | -1.570 | |  | -1.391 |  | | 0.090 |  |
| uku_auto |  | -0.524 | |  | 0.051 | |  | -0.291 |  | | 0.358 |  |
| uku_neuro |  | -0.524 | |  | -0.196 | |  | 0.015 |  | | 0.139 |  |
| uku_psy |  | 0.000 | |  | 0.361 | |  | 0.244 |  | | 0.499 |  |
| uku_sex |  | -0.524 | |  | -1.280 | |  | -1.283 |  | | -0.250 |  |
| whodas_total |  | -0.524 | |  | 0.417 | |  | 0.163 |  | | 0.145 |  |
|  | | | | | | | | | | | | |

**Appendix D**

**Network analysis for diagnosis subgroups**

Here, we conducted an additional network analysis, complementing the main manuscript, to compare the pattern of interactions among disability, quality of life, symptoms, and side effects based on the GASS versus the UKU (Figure 1). To examine diagnosis-specific differences, each network was estimated separately (i.e., stratified) for schizophrenia-spectrum disorders and bipolar disorder. However, given the limited sample size, the estimated networks were fully sparse (100% edge sparsity) for the bipolar subgroup with GASS and for both schizophrenia and bipolar subgroups with UKU, resulting in empty network plots.

Fig. S2 Interactions between disability, quality of life, symptoms and side effects using GASS in schizophrenia spectrum disorders versus bipolar disorder.

*^Network 1 = schizophrenia spectrum disorders; Network 2 = bipolar disorder^*

Table S10. Summary of network structure and node centrality in GASS-based subgroups

| *Summary of Network* | | | | | | | |
| --- | --- | --- | --- | --- | --- | --- | --- |
| Network | | Number of nodes | | Number of non-zero edges | | Sparsity | |
| 1 |  | 8 |  | 20 / 28 |  | 0.286 |  |
| 2 |  | 8 |  | 0 / 28 |  | 1.000 |  |
|  | | | | | | | |

| *Centrality measures per variable* | | | | | | | | | | | | | | | | | |
| --- | --- | --- | --- | --- | --- | --- | --- | --- | --- | --- | --- | --- | --- | --- | --- | --- | --- |
|  | | 1 | | | | | | | | 2 | | | | | | | |
| Variable | | Betweenness | | Closeness | | Strength | | Expected influence | | Betweenness | | Closeness | | Strength | | Expected influence | |
| gass_auto |  | 0.403 |  | 0.610 |  | 1.496 |  | 1.152 |  | 0.000 |  | 0.000 |  | 0.000 |  | 0.000 |  |
| gass_neuro |  | -0.242 |  | 0.399 |  | -0.354 |  | 0.548 |  | 0.000 |  | 0.000 |  | 0.000 |  | 0.000 |  |
| gass_psy |  | -0.242 |  | 0.057 |  | -0.059 |  | -4.460×10^-4^ |  | 0.000 |  | 0.000 |  | 0.000 |  | 0.000 |  |
| gass_sex |  | -0.564 |  | -0.598 |  | -0.870 |  | -0.379 |  | 0.000 |  | 0.000 |  | 0.000 |  | 0.000 |  |
| health_today |  | -0.564 |  | -1.685 |  | -1.033 |  | -1.939 |  | 0.000 |  | 0.000 |  | 0.000 |  | 0.000 |  |
| madrs_tot |  | 2.335 |  | 1.765 |  | 1.578 |  | 1.065 |  | 0.000 |  | 0.000 |  | 0.000 |  | 0.000 |  |
| mrs_tot |  | -0.564 |  | -0.255 |  | -0.501 |  | -0.567 |  | 0.000 |  | 0.000 |  | 0.000 |  | 0.000 |  |
| whodas_total |  | -0.564 |  | -0.293 |  | -0.257 |  | 0.120 |  | 0.000 |  | 0.000 |  | 0.000 |  | 0.000 |  |
|  | | | | | | | | | | | | | | | | | |

Fig. S3 Interactions between disability, quality of life, symptoms and side effects using UKU in schizophrenia spectrum disorders versus bipolar disorder.

*Network 1 = schizophrenia spectrum disorder; Network 2 = bipolar disorder*

Table S11. Summary of network structure and node centrality in UKU-based subgroups

| *Summary of Network* | | | | | | | |
| --- | --- | --- | --- | --- | --- | --- | --- |
| Network | | Number of nodes | | Number of non-zero edges | | Sparsity | |
| 1 |  | 8 |  | 0 / 28 |  | 1.000 |  |
| 2 |  | 8 |  | 0 / 28 |  | 1.000 |  |
|  | | | | | | | |
|  | | | | | | | |
| *Centrality measures per variable* | | | | | | | |

|  | | 1 | | | | | | | | 2 | | | | | | | |
| --- | --- | --- | --- | --- | --- | --- | --- | --- | --- | --- | --- | --- | --- | --- | --- | --- | --- |
| Variable | | Betweenness | | Closeness | | Strength | | Expected influence | | Betweenness | | Closeness | | Strength | | Expected influence | |
| health_today |  | 0.000 |  | 0.000 |  | 0.000 |  | 0.000 |  | 0.000 |  | 0.000 |  | 0.000 |  | 0.000 |  |
| madrs_tot |  | 0.000 |  | 0.000 |  | 0.000 |  | 0.000 |  | 0.000 |  | 0.000 |  | 0.000 |  | 0.000 |  |
| mrs_tot |  | 0.000 |  | 0.000 |  | 0.000 |  | 0.000 |  | 0.000 |  | 0.000 |  | 0.000 |  | 0.000 |  |
| uku_auto |  | 0.000 |  | 0.000 |  | 0.000 |  | 0.000 |  | 0.000 |  | 0.000 |  | 0.000 |  | 0.000 |  |
| uku_neuro |  | 0.000 |  | 0.000 |  | 0.000 |  | 0.000 |  | 0.000 |  | 0.000 |  | 0.000 |  | 0.000 |  |
| uku_psy |  | 0.000 |  | 0.000 |  | 0.000 |  | 0.000 |  | 0.000 |  | 0.000 |  | 0.000 |  | 0.000 |  |
| uku_sex |  | 0.000 |  | 0.000 |  | 0.000 |  | 0.000 |  | 0.000 |  | 0.000 |  | 0.000 |  | 0.000 |  |
| whodas_total |  | 0.000 |  | 0.000 |  | 0.000 |  | 0.000 |  | 0.000 |  | 0.000 |  | 0.000 |  | 0.000 |  |
|  | | | | | | | | | | | | | | | | | |

**Network analyses using side effects total score**

We also ran network analyses using the GASS and UKU global scores. Because our primary objective was to examine side-effect subdomains and their network roles, these global-score results are reported here rather than in the main text.

Fig. S4 Interactions between disability, quality of life, symptoms and side effects using GASS overall score in schizophrenia spectrum disorders versus bipolar disorder

Table S12. Summary of network structure and node centrality in GASS overall score network plot

| *Summary of Network* | | | | | | | | |  |
| --- | --- | --- | --- | --- | --- | --- | --- | --- | --- |
| Number of nodes | | | Number of non-zero edges | | | | Sparsity | |  |
| 5 |  | | 9 / 10 | | |  | 0.100 |  |  |
|  | | | | | | | | |  |
| Centrality measures per variable | | | | | | | | |  |
|  | | Network | | | | | | | |
| Variable | | Betweenness | | Closeness | | Strength | | Expected influence | |
| gass_total |  | -0.447 |  | 0.049 |  | 0.067 |  | 0.635 |  |
| health_today |  | -0.447 |  | 0.086 |  | 0.314 |  | -1.776 |  |
| madrs_tot |  | 1.789 |  | 0.907 |  | 0.735 |  | 0.462 |  |
| mrs_tot |  | -0.447 |  | -1.667 |  | -1.727 |  | 0.342 |  |
| whodas_total |  | -0.447 |  | 0.625 |  | 0.611 |  | 0.337 |  |
|  | | | | | | | | | |

Fig. S5 Interactions between disability, quality of life, symptoms and side effects using UKU overall score in schizophrenia spectrum disorders versus bipolar disorder

Table S13. Summary of network structure and node centrality in UKU overall score network plot

| *Summary of Network* | | | | | | | | |  |
| --- | --- | --- | --- | --- | --- | --- | --- | --- | --- |
| Number of nodes | | | Number of non-zero edges | | | | Sparsity | |  |
| 5 |  | | 6 / 10 | | |  | 0.400 |  |  |
|  | | | | | | | | |  |
| Centrality measures per variable | | | | | | | | |  |
|  | | Network | | | | | | | |
| Variable | | Betweenness | | Closeness | | Strength | | Expected influence | |
| health_today |  | -0.671 |  | 0.026 |  | 0.504 |  | -1.549 |  |
| madrs_tot |  | 1.565 |  | 0.978 |  | 0.952 |  | 1.242 |  |
| mrs_tot |  | -0.671 |  | -1.662 |  | -1.648 |  | 0.202 |  |
| uku_tot |  | 0.447 |  | 0.506 |  | 0.325 |  | 0.125 |  |
| whodas_total |  | -0.671 |  | 0.152 |  | -0.134 |  | -0.020 |  |
|  | | | | | | | | | |

**Appendix E**

Table S14. STROBE Statement completed checklist for cross-sectional, observational studies.

|  | Item No | Recommendation | Article page |
| --- | --- | --- | --- |
| **Title and abstract** | 1 | (*a*) Indicate the study’s design with a commonly used term in the title or the abstract | See “Aim” section in the abstract |
|  |  | (*b*) Provide in the abstract an informative and balanced summary of what was done and what was found | See Abstract in the title page |
| Introduction | | |  |
| Background/rationale | 2 | Explain the scientific background and rationale for the investigation being reported | Page 2-3 |
| Objectives | 3 | State specific objectives, including any prespecified hypotheses | Page 3 |
| Methods | | |  |
| Study design | 4 | Present key elements of study design early in the paper | Page 3-4 |
| Setting | 5 | Describe the setting, locations, and relevant dates, including periods of recruitment, exposure, follow-up, and data collection | Page 3-4 |
| Participants | 6 | (*a*) Give the eligibility criteria, and the sources and methods of selection of participants | Page 3 |
| Variables | 7 | Clearly define all outcomes, exposures, predictors, potential confounders, and effect modifiers. Give diagnostic criteria, if applicable | Page 4-5 |
| Data sources/ measurement | 8 | For each variable of interest, give sources of data and details of methods of assessment (measurement). Describe comparability of assessment methods if there is more than one group | Page 4-5 |
| Bias | 9 | Describe any efforts to address potential sources of bias | Page 3-4 |
| Study size | 10 | Explain how the study size was arrived at | Page 3 |
| Quantitative variables | 11 | Explain how quantitative variables were handled in the analyses. If applicable, describe which groupings were chosen and why | Page 5 |
| Statistical methods | 12 | (*a*) Describe all statistical methods, including those used to control for confounding | Page 5-6 |
|  |  | (*b*) Describe any methods used to examine subgroups and interactions | Appendix C, Appendix D |
|  |  | (*c*) Explain how missing data were addressed | Not applicable (no missing data) |
|  |  | (*d*) If applicable, describe analytical methods taking account of sampling strategy | Not applicable |
|  |  | (*e*) Describe any sensitivity analyses | Not applicable |
| Results | | |  |
| Participants | 13 | (a) Report numbers of individuals at each stage of study—eg numbers potentially eligible, examined for eligibility, confirmed eligible, included in the study, completing follow-up, and analysed | Numbers of recruited (n=111), excluded (n=11), and included (n=100) participants are reported in the primary publication ^1^ |
|  |  | (b) Give reasons for non-participation at each stage | Exclusion was due to missing frequency data required for clinical validation of the Italian GASS scale. |
|  |  | (c) Consider use of a flow diagram | Not applicable, as the participant flow is straightforward and numerically described in text form. |
| Descriptive data | 14 | (a) Give characteristics of study participants (eg demographic, clinical, social) and information on exposures and potential confounders | Appendix A |
|  |  | (b) Indicate number of participants with missing data for each variable of interest | Not applicable (no missing data) |
| Outcome data | 15 | Report numbers of outcome events or summary measures | Appendix B |
| Main results | 16 | (*a*) Give unadjusted estimates and, if applicable, confounder-adjusted estimates and their precision (eg, 95% confidence interval). Make clear which confounders were adjusted for and why they were included | Not applicable |
|  |  | (*b*) Report category boundaries when continuous variables were categorized | Not applicable |
|  |  | (*c*) If relevant, consider translating estimates of relative risk into absolute risk for a meaningful time period | Not applicable |
| Other analyses | 17 | Report other analyses done—eg analyses of subgroups and interactions, and sensitivity analyses | Appendix C, Appendix D |
| Discussion | | |  |
| Key results | 18 | Summarise key results with reference to study objectives | Page 10-11 |
| Limitations | 19 | Discuss limitations of the study, taking into account sources of potential bias or imprecision. Discuss both direction and magnitude of any potential bias | Page 11-12 |
| Interpretation | 20 | Give a cautious overall interpretation of results considering objectives, limitations, multiplicity of analyses, results from similar studies, and other relevant evidence | Page 12 |
| Generalisability | 21 | Discuss the generalisability (external validity) of the study results | Page 12 |
| Other information | | |  |
| Funding | 22 | Give the source of funding and the role of the funders for the present study and, if applicable, for the original study on which the present article is based | Page 13 |

*Reference: von Elm, E., Altman, D. G., Egger, M., Pocock, S. J., Gøtzsche, P. C., Vandenbroucke, J. P., & STROBE Initiative. (2007). The Strengthening the Reporting of Observational Studies in Epidemiology (STROBE) statement: Guidelines for reporting observational studies. Lancet (London, England), 370(9596), 1453–1457. https://doi.org/10.1016/S0140-6736(07)61602-X*

^1^ Rodolico, A., Concerto, C., Ciancio, A., Siafis, S., Fusar-Poli, L., Romano, C. B., Scavo, E. V., Petralia, A., Salomone, S., Signorelli, M. S., Leucht, S., & Aguglia, E. (2022). Validation of the Glasgow Antipsychotic Side-Effect Scale (GASS) in an Italian Sample of Patients with Stable Schizophrenia and Bipolar Spectrum Disorders. Brain sciences, 12(7), 891. <https://doi.org/10.3390/brainsci12070891>
